# High-flow nasal oxygen therapy via a single-prong cannula interface during bronchoscopy in patients with acute respiratory failure: a two-center, open-label, randomized controlled trial

**DOI:** 10.1016/j.aicoj.2026.100081

**Published:** 2026-05-12

**Authors:** Rui Wang, Yu Zhao, Ning Lan, Wancong Wang, Xiao Tang, Ting Li, Xiaoqin Liu, Haichao Li, Li Meng, Zhi Xu, Li Wang, Bing Sun, Guifen Gan

**Affiliations:** a Department of Respiratory and Critical Care Medicine, Beijing Institute of Respiratory Medicine and Beijing Chao-Yang Hospital, Capital Medical University, No. 8 Gongren Tiyuchang Nanlu, Chaoyang District, Beijing, China; b Xi'an Center for Disease Control and Prevention, No. 599 Yanta District, Xi'an, Shaanxi Province, China; c Department of Critical Care Medicine, Affiliated Hospital of Qinghai University, No. 29 Tongren Road, Chengdong District, Xining, Qinghai Province, China

**Keywords:** High-flow nasal oxygen therapy, Single-prong cannula interface, Flexible bronchoscopy, Acute respiratory failure, Standard oxygen therapy

## Abstract

**Background:**

Flexible bronchoscopy (FB) is commonly performed in patients with acute respiratory failure (ARF), but the procedure can exacerbate hypoxemia and increase the need for respiratory support. The impact of high-flow nasal oxygen (HFNO) therapy during nasal FB in patients with ARF remains uncertain, as prior studies have focused primarily on oxygenation rather than clinical outcomes. We aimed to evaluate whether HFNO therapy via a single-prong cannula interface (HFNO-SPC) could reduce the need for respiratory support escalation within 24 h after FB compared to standard oxygen therapy (SOT).

**Methods:**

We conducted a two-center, open-label, randomized controlled trial comparing HFNO-SPC to SOT in patients undergoing FB. The primary outcome was escalation of respiratory support within 24 h after FB, defined as the requirement for invasive mechanical ventilation (IMV), noninvasive ventilation (NIV), or HFNO, or as increases in support parameters without changing the mode of respiratory support. The secondary outcome was a hierarchical composite of these escalation events. Electrical impedance tomography (EIT) monitoring was performed during FB to assess tidal volume and end-expiratory lung volume changes.

**Results:**

A total of 160 patients were randomized to either the HFNO-SPC group (n = 80) or the SOT group (n = 80), and all were included in the intention-to-treat analysis. HFNO-SPC significantly reduced the incidence of respiratory support escalation compared to SOT (15.0% vs. 33.8%, *P* = 0.006). Hierarchical analysis of the composite secondary outcome supported the primary findings (31.6% vs 12.1%, *P* = 0.004). Additionally, HFNO-SPC resulted in a lower intubation rate within 24 h (7.5% vs. 20.0%, *P* = 0.022). EIT measurements showed smaller reductions in tidal impedance variation (TIV) during FB and less pronounced changes in end-expiratory lung impedance (ΔEELI) during and after FB in the HFNO-SPC group.

**Conclusions:**

In patients with ARF, HFNO-SPC significantly reduced the proportion of patients requiring respiratory support escalation within 24 h after FB compared with SOT. This finding was consistent across a hierarchical analysis of clinically ranked outcomes.

**Trial Registration:**

ClinicalTrials.Gov: NCT 05759832. Registered 27 February 2023.

## Background

Flexible bronchoscopy (FB) is a widely used diagnostic and therapeutic procedure in patients with acute respiratory failure (ARF) [1]. Despite the use of supplemental oxygen during the procedure, some patients may experience hypoxemia, leading to deterioration of pre-existing respiratory failure and the need for escalated respiratory support or even endotracheal intubation [2,3]. In non-intubated patients with ARF, previous randomized controlled trials (RCTs) have demonstrated that noninvasive ventilation (NIV) can effectively maintain adequate oxygenation during FB [[4], [5], [6]]. However, NIV may not be suitable for all patients due to poor patient-ventilator synchrony during the procedure, which may reduce patient tolerability [7]. This limitation has led to growing interest in high‐flow nasal oxygen (HFNO) therapy as an alternative oxygenation strategy.

HFNO delivers humidified air and oxygen at flow rates up to 60 L/min, promoting pharyngeal dead space washout and generating positive expiratory airway pressure, which subsequently increases end-expiratory lung volume [8,9]. Several RCTs have demonstrated that HFNO therapy can effectively reduce the occurrence of hypoxemia during FB in post-lung transplant recipients, patients with chronic obstructive pulmonary disease (COPD), and other high-risk populations [[10], [11], [12]]. However, HFNO therapy has been reported to provide less stable oxygenation during FB compared to NIV in patients with ARF, despite being associated with fewer cases requiring endotracheal intubation [4,5]. One possible explanation for this finding is that FB was typically performed via the oral route in previous studies, which may have compromised some of the physiological benefits of HFNO therapy. During open-mouth breathing, the positive expiratory airway pressure generated by HFNO is significantly reduced, and additional ambient air may be entrained, thereby diminishing effective flow rates and oxygen delivery [13,14]. In addition, these studies primarily focused on peripheral oxygen saturation (SpO_2_) in patients with ARF, while RCTs evaluating the impact of HFNO therapy as an oxygenation strategy during nasal FB on clinical outcomes are still lacking.

Beyond oxygenation alone, FB may induce transient airway obstruction, increased respiratory effort, and loss of end-expiratory lung volume (EELV), leading to physiological changes that are not fully captured by routine vital signs alone. Electrical impedance tomography (EIT) provides bedside, noninvasive monitoring of tidal volume and EELV, thereby better characterizing these dynamic respiratory alterations that may contribute to post-FB clinical deterioration [15].

We hypothesized that HFNO therapy applied during FB may improve patient prognosis compared to standard oxygen therapy (SOT). The use of large-bore nasal prongs as the standard interface in HFNO therapy poses a significant challenge to the performance of nasal FB. To address this, we developed a modified HFNO interface using a single-prong nasal cannula. In our previously published study involving an unselected population without respiratory failure, HFNO via this interface significantly reduced the incidence of hypoxemia compared to low-flow nasal oxygen (LFNO) therapy [16]. To extend our previous work, we conducted a two-center, open-label, RCT to investigate whether HFNO therapy via a single-prong cannula interface (HFNO-SPC) would reduce the need for respiratory support escalation within 24 h after FB compared with SOT in patients with ARF. In addition, to explore the physiological effects of different oxygenation strategies during FB, we performed exploratory EIT measurements to monitor changes in tidal volume and EELV during and after the procedure.

## Methods

### Trial design and oversight

This two-center, open-label, RCT was conducted in the intensive care units (ICUs) of two tertiary care hospitals in China: Beijing Chao-Yang Hospital and Qinghai University Affiliated Hospital. The trial was conducted in accordance with the Declaration of Helsinki and Good Clinical Practice guidelines, approved by the ethics committees of both participating centers, and registered at ClinicalTrials.gov (NCT05759832). Written informed consent was obtained from the patients themselves or their legal guardians. The study protocol and statistical analysis plan are available in the supplementary material.

The trial was undertaken at two centers and coordinated by a single principal investigator. At each center, a senior physician and two respiratory therapists were responsible for daily patient screening and trial enrollment, monitoring protocol adherence, and completing the case report forms. All investigators had extensive experience in the use of HFNO therapy, NIV, invasive mechanical ventilation (IMV), and EIT, and all received standardized training on the study protocol.

### Patients

We included patients who met the following criteria: [1] age ≥18 years [2]; respiratory failure defined as a ratio of the partial pressure of arterial oxygen to the fraction of inspired oxygen (PaO_2_/FiO_2_) <300 mm Hg; and [3] a clinical indication for FB to diagnose or treat pulmonary disease. Patients were excluded if they met any of the following criteria: (1) already intubated or tracheostomized; (2) required immediate endotracheal intubation; (3) PaO_2_/FiO_2_ <150 mm Hg; (4) platelet count <60 × 10^9^/L; (5) history of myocardial infarction within the past 6 weeks; (6) nasopharyngeal obstruction or blockage; (7) presence of chest skin lesions contraindicating the application of EIT; and (8) intolerance to HFNO therapy.

### Patient and public involvement

Patients were not involved in the design, conduct or reporting of the study.

### Randomization and blinding

Eligible patients were randomly assigned in a 1:1 ratio to receive either HFNO-SPC or SOT. Randomization was stratified by center and conducted using a computer-generated sequence with variable block sizes. Allocation concealment was maintained using sequentially numbered, opaque, sealed envelopes.

Blinding of participants and investigators was not feasible due to the visible nature of HFNO delivery; therefore, the study was conducted as an open-label trial. Data analysis was performed by an independent statistician who was not involved in the trial.

### Interventions

In the HFNO-SPC group, high-flow respiratory support was delivered using AIRVO 2 devices (Fisher & Paykel Healthcare, Auckland, New Zealand) via a single-prong nasal cannula interface. The size of the nasal cannula was selected based on the patient’s nostril size, with the cannula diameter ideally occupying approximately 50%–70% of the nostril diameter. The FiO_2_ was initially set at 0.8 and could be adjusted up to 1.0 during FB to maintain SpO_2_ above 90%. The flow rate and temperature were set at 60 L/min and 34 °C, respectively.

In the SOT group, oxygen was delivered via a non-rebreathing reservoir mask (Intersurgical Ltd., Wokingham, Berkshire, UK). The initial flow rate was set at 10 L/min and could be increased up to 15 L/min during FB to maintain SpO_2_ above 90%.

### Procedures

The following patient characteristics were collected at the time of randomization: age, sex, body mass index, Acute Physiology and Chronic Health Evaluation II (APACHE II) score, Sequential Organ Failure Assessment (SOFA) score, smoking history, underlying comorbidities, indication for FB, type of respiratory support before FB, vital signs, arterial blood gas parameters, and laboratory test results.

After randomization, a properly sized 16-electrode EIT belt was placed around the patient’s chest, just below the axillae at the level of the fourth to fifth intercostal spaces. The belt position was marked to prevent displacement during the study. EIT data were acquired using a PulmoVista® 500 device (Dräger, Lübeck, Germany) at a sampling rate of 20 Hz and stored for offline analysis.

Arterial blood gas samples and invasive blood pressure measurements were obtained via a radial or femoral arterial catheter. Electrocardiogram (ECG), respiratory rate, heart rate, and SpO_2_ were continuously monitored using a bedside ECG monitor.

All bronchoscopies were performed by experienced pulmonologist and intensivist. For local anesthesia, 5 mL of 2% lidocaine was nebulized into the nasal cavity and pharyngeal mucosa. All patients were allowed to rest for approximately 5 min to ensure the anesthetic effect had taken full effect. Patients in both groups received supplemental oxygen via the assigned oxygenation method for 5 min before sedation. Conscious sedation was achieved with intravenous midazolam at 0.05 to 0.1 mg/kg and propofol up to 1.0 mg/kg, each titrated to the minimum effective dose based on patient response. Sedation depth was clinically adjusted to achieve light to moderate sedation, corresponding approximately to a Richmond Agitation-Sedation Scale (RASS) score between −1 and −2. FB was performed using an Olympus BF-260 and BF-1T260 bronchoscope (Olympus Corporation, Tokyo, Japan) via the nasal route with the patient in the supine position. Patients were instructed to keep their mouths closed throughout the procedure whenever possible. When necessary, simple manual support (e.g., gentle jaw support) was applied to minimize mouth opening. Once the FB was advanced into the trachea, lidocaine was sprayed onto the carina. The bronchial tree was then systematically examined, and the bronchoscope was wedged into the appropriate segmental bronchus. Bronchoalveolar lavage (BAL) was performed using normal saline, instilled in 20 mL aliquots followed by gentle suction. This process was repeated 3–5 times depending on the suspected disease category and the patient’s clinical condition. BAL fluid was sent for cytological and/or microbiological analysis. The decision to perform bronchial brushing, endobronchial biopsy, or transbronchial lung biopsy was not part of the study protocol and was therefore left to the discretion of the bronchoscopist. The duration of FB was defined as the time from insertion of the bronchoscope into the nasal cavity to its complete withdrawal from the airway.

FB interruption was defined as temporary discontinuation of the procedure when SpO_2_ persistently decreased below 90%, at the discretion of the bronchoscopist. The procedure could be resumed once SpO_2_ recovered to ≥90%. When SpO_2_ failed to improve despite escalation of the assigned oxygenation method to its maximum support level, the procedure was definitively terminated and advanced airway management was initiated as clinically indicated, including the use of an oropharyngeal airway, laryngeal mask, or endotracheal intubation. After FB, patients continued on the assigned oxygenation method until clinically stable, after which the clinical team determined whether to resume the pre-FB respiratory support. Temporary changes in oxygen supplementation or ventilatory support during FB or within 30 min after FB were not classified as escalation of respiratory support in this study, as these changes were considered transient, procedure-related adjustments rather than clinically meaningful deterioration.

Vital signs and EIT data were collected at five predefined time points: T0, before FB; T1, upon insertion of the FB into the nasal cavity; T2, at the end of the procedure; T3, 10 min after FB; and T4, 2 h after FB. Arterial blood gas samples were obtained at T0 and T4 for analysis.

EIT recordings were analyzed offline by averaging five consecutive respiratory cycles selected from a visually stable segment at predefined time points to compute:

(1) Tidal impedance variation (TIV) was calculated as a quantitative estimate of tidal volume [15]. To enable the assessment of lung volume changes using EIT, the TIV at T0 was calibrated against the tidal volume measured by the NIV device (Philips Respironics V60; Philips Respironics, Murrysville, PA, USA). NIV was delivered using a Mirage Quattro™ oronasal mask (ResMed, San Diego, CA, USA).

(2) Changes in end-expiratory lung impedance (ΔEELI), which reflect variations in EELV, were calculated at T1, T2, T3, and T4 relative to T0 [17] (Section S1).

FB-associated adverse events were also recorded, with detailed definitions provided in the supplementary material (Section S2).

### Study outcomes

The primary outcome was the need for respiratory support escalation within 24 h after FB, defined as meeting any of the following criteria compared with the level of respiratory support before FB: (1) escalation to IMV; (2) escalation to NIV; (3) escalation to HFNO therapy; or (4) an increase in support parameters without changing the mode of respiratory support, including a greater than 20% increase in inspiratory positive airway pressure (IPAP), expiratory positive airway pressure (EPAP), or FiO_2_ for patients on NIV; a greater than 20% increase in flow rate or FiO_2_ for those on HFNO therapy; and a more than 50% increase in oxygen flow rate for those on LFNO therapy [2,3]. The secondary outcome was a hierarchical composite outcome, assessed in a fixed sequence: escalation to IMV, escalation to NIV, escalation to HFNO therapy, and an increase in support parameters without changing the mode of respiratory support. Other outcomes included the need for intubation within 24 h, 7 days, and 28 days after FB; the lowest SpO_2_ recorded during FB; the number of patients requiring interruption of FB; the duration of FB; ICU and hospital length of stay; and mortality at 28, 60, and 90 days. Respiratory support escalation strategy are provided in the supplementary material (Section S3).

### Statistical analysis

The sample size was calculated based on the primary hypothesis that HFNO-SPC would reduce the incidence of respiratory support escalation within 24 h after FB compared with SOT in patients with ARF. Based on previous published studies, it was estimated that approximately 35% of patients in the SOT group would require escalation of respiratory support [2]. The assumption that HFNO-SPC could reduce this rate to 15% was informed by our pilot data. Under this hypothesis, a total of 144 patients would be required to detect this difference with 80% power at a two-sided alpha level of 0.05. To account for 10% potential dropouts, the final enrollment target was set at 160 patients.

The primary analyses were performed using an intention-to-treat principle. Per-protocol analyses were also conducted. Categorical variables, including the primary and other dichotomous outcomes, were compared using either the chi-square test or Fisher’s exact test, as appropriate. Continuous variables were compared using Student’s t-test or the Mann–Whitney U test, as appropriate. The secondary outcome of respiratory support escalation within 24 h after FB was analyzed using the unmatched win ratio method. This composite outcome consisted of four hierarchically ordered components based on clinical relevance: (1) escalation to IMV, (2) escalation to NIV, (3) escalation to HFNO therapy, and (4) an increase in support parameters without changing the mode of respiratory support. Absolute differences in proportions and corresponding 95% confidence intervals (CIs) were estimated using either the Wald asymptotic method or the Miettinen-Nurminen method. For continuous outcomes, differences in median values and their 95% CIs were estimated using 5,000 bootstrap resamples. Univariable logistic regression or Cox proportional hazards models were used to assess the effect of HFNO-SPC compared with SOT on the primary, secondary, and other outcomes.

The cumulative incidence of time-to-event outcomes was estimated using the Kaplan–Meier method and compared using the log-rank test. These outcomes included respiratory support escalation within 24 h, endotracheal intubation within 24 h and within 7 days, and 28-day mortality, all following FB. The overall time course of vital signs and EIT measurements was compared using a two-way repeated-measures analysis of variance. To illustrate the association between ΔEELI at T2, T3, and T4 and the predicted probability of respiratory support escalation within 24 h after FB, logistic regression models with restricted cubic splines were used to estimate adjusted probabilities with 95% CIs. Prespecified subgroup analyses were conducted based on study center, age, sex, APACHE II score, immunocompromised status, respiratory support, and PaO_2_/FiO_2_ ratio at randomization, as well as ΔEELI at T2 and the amount of BAL fluid instilled.

Missing data were not imputed. All P values were two-sided, and values less than 0.05 were considered statistically significant. Data analyses were performed using R version 4.4.2 (R Foundation for Statistical Computing, Vienna, Austria) and GraphPad Prism version 9 (GraphPad Software Inc., San Diego, CA, USA).

## Results

### Patients

**Fig. 1:**
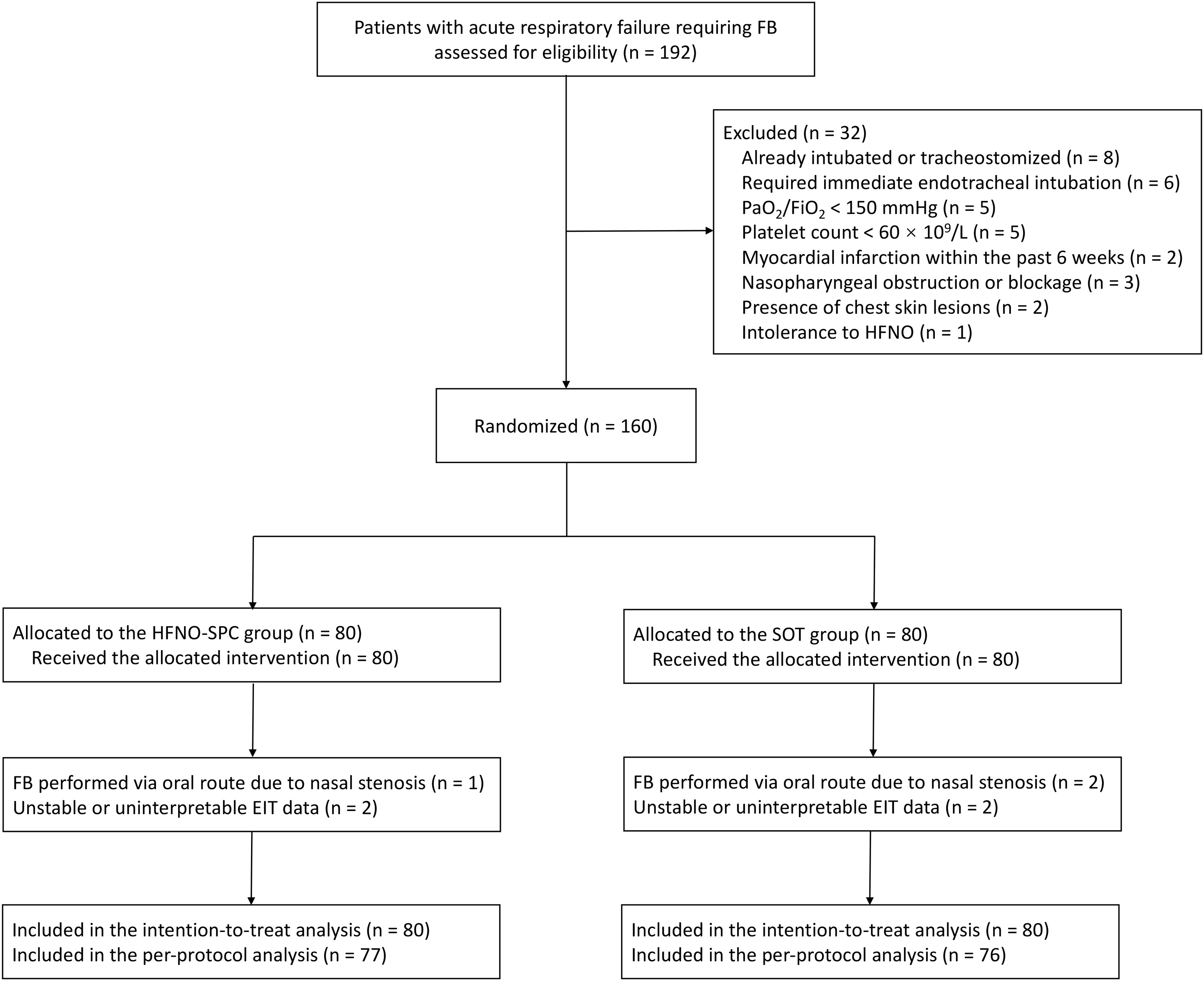
Flow diagram for the trial. FB flexible bronchoscopy, PaO_2_/FiO_2_ the ratio of the partial pressure of arterial oxygen to the fraction of inspired oxygen, HFNO high-flow nasal oxygen, HFNO-SPC HFNO therapy via a single-prong cannula interface, SOT standard oxygen therapy.

From April 1, 2023, to June 30, 2025, 192 patients with acute respiratory failure were assessed for eligibility. Thirty-two patients were excluded, and 160 were randomized to the HFNO-SPC group (n = 80) or the SOT group (n = 80) (Fig. 1). Of these, 88 patients were enrolled at Beijing Chao-Yang Hospital and 72 at Qinghai University Affiliated Hospital. Baseline characteristics were similar between the two groups (Table 1). The mean age of patients was 58 ± 17 years, and 103 patients (64.4%) were male. The most common indication for FB was severe community-acquired pneumonia, observed in 82 patients (52.3%). Before FB, 11 patients (6.9%) received LFNO therapy, 112 (70.0%) received HFNO therapy, and 37 (23.1%) received NIV. Baseline laboratory test results also showed no significant differences between the two groups (Table S1).

**Table 1:** Characteristics of patients at randomization.

Characteristics	HFNO-SPC group (n = 80)	SOT group (n = 80)
Age, years	58 ± 17	57 ± 16
Male, no. (%)	53 (66.3)	50 (62.5)
Body mass index, kg/m^2^	24.6 ± 3.8	24.6 ± 4.3
APACHE II score	14 (10–19)	14 (9–20)
SOFA score	6 (4–8)	6 (5–7)
Smoking history, no. (%)		
Ever smoked	27 (33.8)	32 (40)
Currently smoke	53 (66.2)	48 (60)
Comorbidities, no. (%)		
Immunocompromised	11 (13.8)	8 (10.0)
COPD or asthma	8 (10.0)	7 (8.8)
Coronary artery disease	9 (11.3)	13 (16.3)
Hypertension	31 (38.8)	36 (45.0)
Diabetes mellitus	14 (17.5)	15 (18.8)
Chronic renal insufficiency	8 (10.0)	5 (6.3)
Cerebrovascular disease	3 (3.8)	4 (5.0)
Indication for FB, no. (%)		
Severe community-acquired pneumonia	44 (55.0)	38 (47.5)
Suspected hospital-acquired pneumonia	5 (6.3)	8 (10.0)
Pneumonia in immunocompromised host	10 (12.5)	7 (8.8)
Interstitial lung disease	16 (20.0)	18 (22.5)
Suspected lung cancer	4 (5.0)	5 (6.3)
Hemoptysis	1 (1.3)	4 (5.0)
Respiratory support before FB, no. (%)		
LFNO therapy	4 (5.0)	7 (8.8)
HFNO therapy	54 (67.5)	58 (72.5)
NIV	22 (27.5)	15 (18.8)
Vital signs		
Temperature, °C	36.9 (36.6–37.2)	36.9 (36.5–37.3)
Respiratory rate, beats/min	24 ± 4	24 ± 4
Heart rate, beats/min	83 ± 15	85 ± 15
SpO_2_, %	96 (94–97)	96 (94–97)
Mean arterial pressure, mmHg	89 ± 16	89 ± 19
Arterial blood gas		
pH	7.45 (7.41–7.49)	7.45 (7.40–7.48)
PaO_2_, mmHg	74.4 ± 9.7	74.1 ± 7.8
PaCO_2_, mmHg	34.9 ± 5.3	36.1 ± 6.0
HCO_3_^−^, mmol/L	26.4 ± 4.5	25.6 ± 4.3
PaO_2_/FiO_2_, mmHg	180.5 (160.0–213.3)	188.5 (160.5–219.5)

HFNO-SPC high-flow nasal oxygen therapy via a single-prong cannula interface, SOT standard oxygen therapy, APACHE II Acute Physiology and Chronic Health Evaluation II, SOFA sequential organ failure assessment, COPD chronic obstructive pulmonary disease, FB flexible bronchoscopy, LFNO low-flow nasal oxygen, NIV noninvasive ventilation, SpO_2_ peripheral oxygen saturation, PaO_2_ partial pressure of arterial oxygen, PaCO_2_ partial pressure of arterial carbon dioxide, HCO_3_^−^ bicarbonate, FiO_2_ fraction of inspired oxygen, PaO_2_/FiO_2_ the ratio of the partial pressure of arterial oxygen to the fraction of inspired oxygen.

### Primary and secondary outcomes

**Fig. 2:**
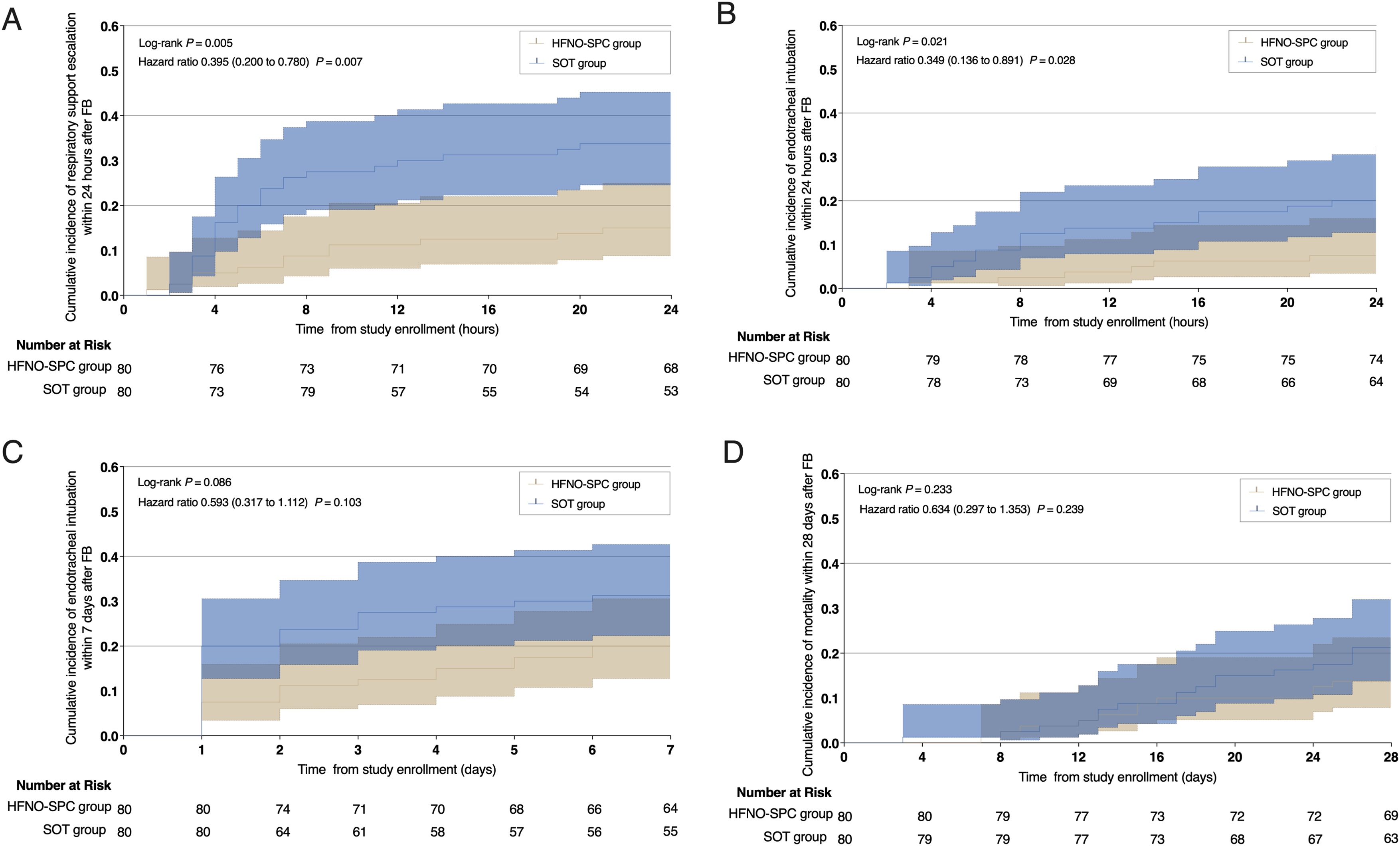
Kaplan-Meier curves for cumulative incidence of clinical outcomes after FB, including respiratory support escalation within 24 h (A), endotracheal intubation within 24 h (B), endotracheal intubation within 7 days (C), and mortality within 28 days (D). FB flexible bronchoscopy, HFNO-SPC high-flow nasal oxygen therapy via a single-prong cannula interface, SOT standard oxygen therapy.

The primary outcome of respiratory support escalation within 24 h after FB occurred in 12 of 80 patients (15.0%) in the HFNO-SPC group and 27 of 80 patients (33.8%) in the SOT group (absolute difference, −18.8% [95% CI, −31.7% to −5.8%], *P* = 0.006; hazard ratio, 0.395 [95% CI, 0.200 to 0.780]) (Table 2 and Fig. 2). In the prespecified hierarchical testing of secondary endpoints, respiratory support escalation within 24 h after FB was analyzed using the win ratio approach to reflect the clinical priority of each component. The HFNO-SPC group was associated with significantly more wins compared with the SOT group (31.6% vs 12.1%, *P* = 0.004; win ratio 2.623 [95% CI, 1.360–5.056]) (Table 2, Table S2, and Figure S1). The analysis of the primary and secondary outcomes in the per-protocol population was consistent with the primary analysis (Table S3 and Figure S2).

**Table 2:** Clinical outcomes according to study group.

Outcomes	HFNO-SPC group (n = 80)	SOT group (n = 80)	Mean, median, or risk difference, (95% CI)	Relative difference, (95% CI)	*P*
Primary outcome					
Respiratory support escalation within 24 h post-FB, no. (%)	12 (15.0)	27 (33.8)	−18.8 (−31.7 to −5.8)^a^	HR, 0.395 (0.200 to 0.780)	0.006^b^
Secondary outcome					
Respiratory support escalation within 24 h post-FB tested in prespecified fixed sequence, no. (%)	2022 (31.6)	771 (12.1)	19.5 (5.2–33.9)	WR, 2.623 (1.360–5.056)	0.004^c^
Other outcomes					
Intubation within 24 h post-FB, no. (%)	6 (7.5)	16 (20.0)	−12.5 (−23.6 to −1.9)^a^	HR, 0.349 (0.136 to 0.891)	0.022^b^
Intubation within 7d post-FB, no. (%)	16 (20.0)	25 (31.3)	−11.2 (−24.6 to 2.4)^a^	HR, 0.593 (0.317–1.112)	0.103^b^
Intubation within 28d post-FB, no. (%)	19 (23.8)	27 (33.8)	−10.0 (−23.9 to 3.9)^a^	HR, 0.647 (0.359–1.164)	0.162^b^
Lowest SpO_2_ during FB, %	90 (88–92)	86 (83–88)	4.0 (3.0–5.0)^d^	N/A^e^	< 0.001^f^
Number of patients with interrupted FB, no. (%)	37 (46.3)	52 (65.0)	−18.8 (−33.9 to −3.6)^g^	OR, 0.463 (0.243 to 0.870)	0.017^b^
Duration of FB, min	10.4 ± 4.7	12.1 ± 4.7	−1.736 (−3.205 to −0.267)	HR, 1.338 (0.978–1.829)	0.021^h^
ICU length of stay, days	13 (10–20)	17 (11–23)	−3.500 (−6.000 to 0.000)^d^	HR, 1.167 (0.854–1.593)	0.142^f^
Hospital length of stay, days	19 (15–24)	20 (17–26)	−1.000 (−4.000–2.000)^d^	HR, 1.086 (0.793–1.486)	0.317^f^
28-day mortality, no. (%)	11 (13.8)	17 (21.3)	−7.5 (−19.5 to 4.4)^a^	HR, 0.634 (0.297–1.353)	0.212^b^
60-day mortality, no. (%)	15 (18.8)	22 (27.5)	−8.8 (−21.8 to 4.4)^a^	HR, 0.653 (0.339–1.259)	0.189^b^
90-day mortality, no. (%)	17 (21.3)	23 (28.8)	−7.5 (−20.9 to 5.9)^g^	HR, 0.704 (0.376–1.318)	0.273^b^

HFNO-SPC high-flow nasal oxygen therapy via a single-prong cannula interface, SOT standard oxygen therapy, FB flexible bronchoscopy, SpO_2_ peripheral oxygen saturation, ICU intensive care unit, HR, hazard ratio, WR win ratio, N/A not applicable, OR odds ratio.

a The 95% confidence interval for the rate difference was estimated using the Miettinen–Nurminen method.

b *P* values were derived from chi-square tests.

c Secondary outcomes defined per statistical analysis plan.

d The 95% confidence interval for the median difference was estimated based on 5000 bootstrap samples.

e This measure is not applicable to continuous outcomes.

f *P* values were derived from Mann–Whitney U tests.

g The 95% confidence interval for the rate difference was estimated using the Wald method.

h *P* values were derived from t tests.

**Fig. 3:**
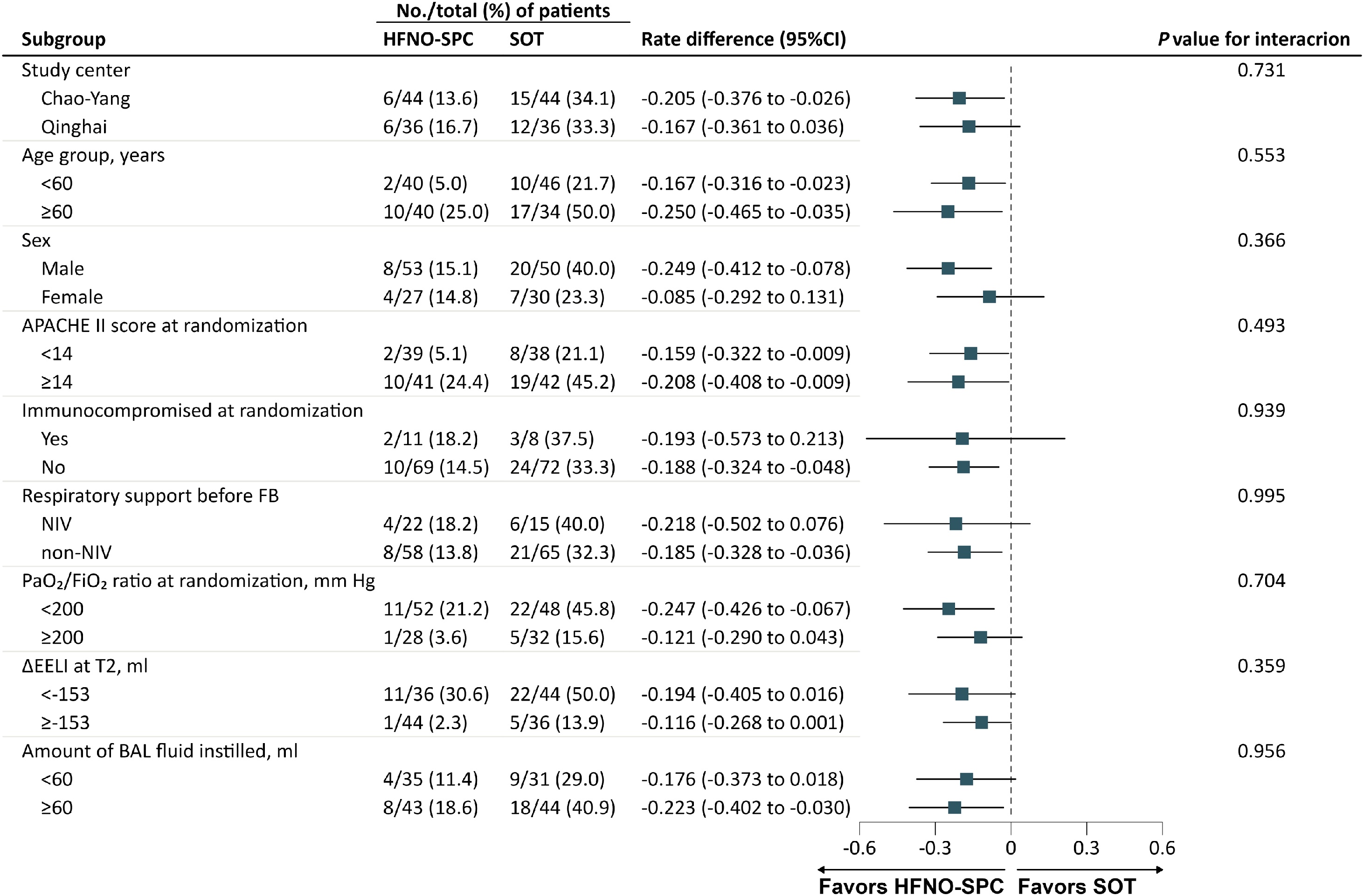
Subgroup analyses for the primary outcome of respiratory support escalation within 24 h after FB. FB flexible bronchoscopy, HFNO-SPC high-flow nasal oxygen therapy via a single-prong cannula interface, SOT standard oxygen therapy, APACHE II Acute Physiology and Chronic Health Evaluation II, NIV noninvasive ventilation, PaO_2_/FiO_2_ the ratio of the partial pressure of arterial oxygen to the fraction of inspired oxygen, ΔEELI the changes in end-expiratory lung impedance, BAL bronchoalveolar lavage.

There was no significant difference in treatment effect across the prespecified subgroups, including study center, age, sex, APACHE II score, immunocompromised status, respiratory support, and PaO_2_/FiO_2_ ratio at randomization, as well as ΔEELI at T2 and the amount of BAL fluid instilled (Fig. 3).

### Other outcomes

Among the other outcomes, the incidence of intubation within 24 h post-FB was significantly lower in the HFNO-SPC group than in the SOT group (7.5% vs. 20.0%, absolute difference, −12.5% [95% CI, −23.6% to −1.9%], *P* = 0.022; hazard ratio, 0.349 [95% CI, 0.136−0.891]) (Table 2 and Fig. 2). The lowest SpO_2_ during FB was significantly higher in the HFNO-SPC group (90% vs. 86%, *P* < 0.001). Furthermore, fewer patients in the HFNO-SPC group experienced FB interruption (46.3% vs. 65.0%, *P* = 0.017), and the procedure duration was also shorter (10.4 ± 4.7 min vs. 12.1 ± 4.7 min, *P* = 0.021) (Table 2).

No significant differences between groups were observed for intubation within 7 days post-FB or for 28-day mortality based on Kaplan−Meier analysis (*P* = 0.086 and *P* = 0.233, respectively) (Fig. 2). Clinical characteristics of patients intubated within 24 h after bronchoscopy are summarized in Table S4.

### Time-course of vital signs, EIT, and arterial blood gases

Vital signs showed dynamic changes over time in both groups (Table 3 and Figure S3). Although both groups experienced a mild drop in SpO_2_ during the procedure, SpO_2_ levels returned to baseline by T4. Additionally, respiratory rate increased during FB in both groups, with a slightly greater increase observed in the SOT group (*P* = 0.077). In the SOT group, heart rate and mean arterial pressure increased transiently during FB and then decreased thereafter, while remaining relatively more stable in the HFNO-SPC group (*P* = 0.014 and *P* < 0.001, respectively).

**Table 3:** Comparison of vital signs and EIT measurements between the HFNO-SPC and SOT groups over time.

Variables	Group	T0	T1	T2	T3	T4	*P*
Vital signs (n = 80/80)							
SpO_2_, %	HFNO-SPC	96 (95–98)	96 (94–97)	94 (91–96)	95 (93–97)	96 (95–98)	< 0.001*^a^*
	SOT	96 (95–98)	96 (95–97)	92 (88–94)	94 (92–96)	97 (95–98)	< 0.001*^a^*
	*P^c^*	0.575	0.341	< 0.001	0.062	0.800	0.016*^b^*
Respiratory rate, beats/min	HFNO-SPC	25 ± 6	25 ± 7	27 ± 5	27 ± 5	26 ± 6	0.136*^a^*
	SOT	25 ± 6	26 ± 6	29 ± 6	28 ± 5	26 ± 5	< 0.001*^a^*
	*P^c^*	0.838	0.281	0.006	0.351	0.502	0.077*^b^*
Heart rate, beats/min	HFNO-SPC	84 ± 13	90 ± 13	94 ± 14	90 ± 12	86 ± 11	< 0.001*^a^*
	SOT	85 ± 13	92 ± 13	101 ± 14	94 ± 13	89 ± 14	< 0.001*^a^*
	*P^c^*	0.555	0.159	< 0.001	0.062	0.147	0.014*^b^*
Mean arterial pressure, mmHg	HFNO-SPC	90 ± 12	93 ± 12	95 ± 15	87 ± 15	88 ± 10	< 0.001*^a^*
	SOT	91 ± 10	96 ± 12	106 ± 19	93 ± 12	90 ± 10	< 0.001*^a^*
	*P^c^*	0.370	0.204	< 0.001	0.010	0.138	< 0.001*^b^*
EIT measurements (n = 78/78)							
TIV, ml	HFNO-SPC	492 (401–625)	481 (385–629)	430 (350–570)	456 (352–591)	460 (376–596)	< 0.001*^a^*
	SOT	491 (402–585)	477 (380–570)	390 (299–385)	422 (338–531)	458 (374–543)	< 0.001*^a^*
	*P^c^*	0.693	0.467	0.024	0.148	0.475	0.156*^b^*
ΔEELI, ml	HFNO-SPC	0 (0 to 0)	−24 (−35 to −10)	−123 (−230 to −31)	−97 (−189 to −37)	−67 (−96 to −22)	< 0.001*^a^*
	SOT	0 (0 to 0)	−29 (−40 to −12)	−216 (−320 to −54)	−141 (−248 to −62)	−85 (−130 to 31)	< 0.001*^a^*
	*P^c^*	–	0.139	0.002	0.008	0.005	< 0.001*^b^*

EIT electrical impedance tomography, HFNO-SPC high-flow nasal oxygen therapy via a single-prong cannula interface, SOT standard oxygen therapy, SpO_2_ peripheral oxygen saturation, TIV tidal impedance variation, ΔEELI the changes in end-expiratory lung impedance.

*p^a^* for overall comparisons of differences in each group over time.

*p^b^* for overall comparisons of differences between groups over time.

*p^c^* for comparisons of differences between groups at each time point.

**Fig. 4:**
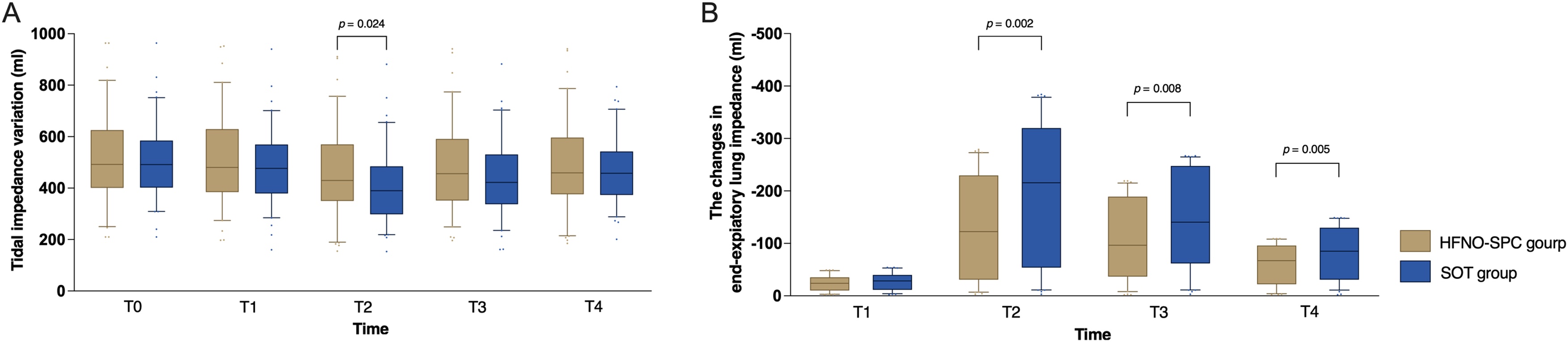
Comparison of tidal impedance variation (A) and the changes in end-expiratory lung impedance (B) between the HFNO-SPC group and the SOT group at predefined time points. HFNO-SPC high-flow nasal oxygen therapy via a single-prong cannula interface, SOT standard oxygen therapy.

**Fig. 5:**
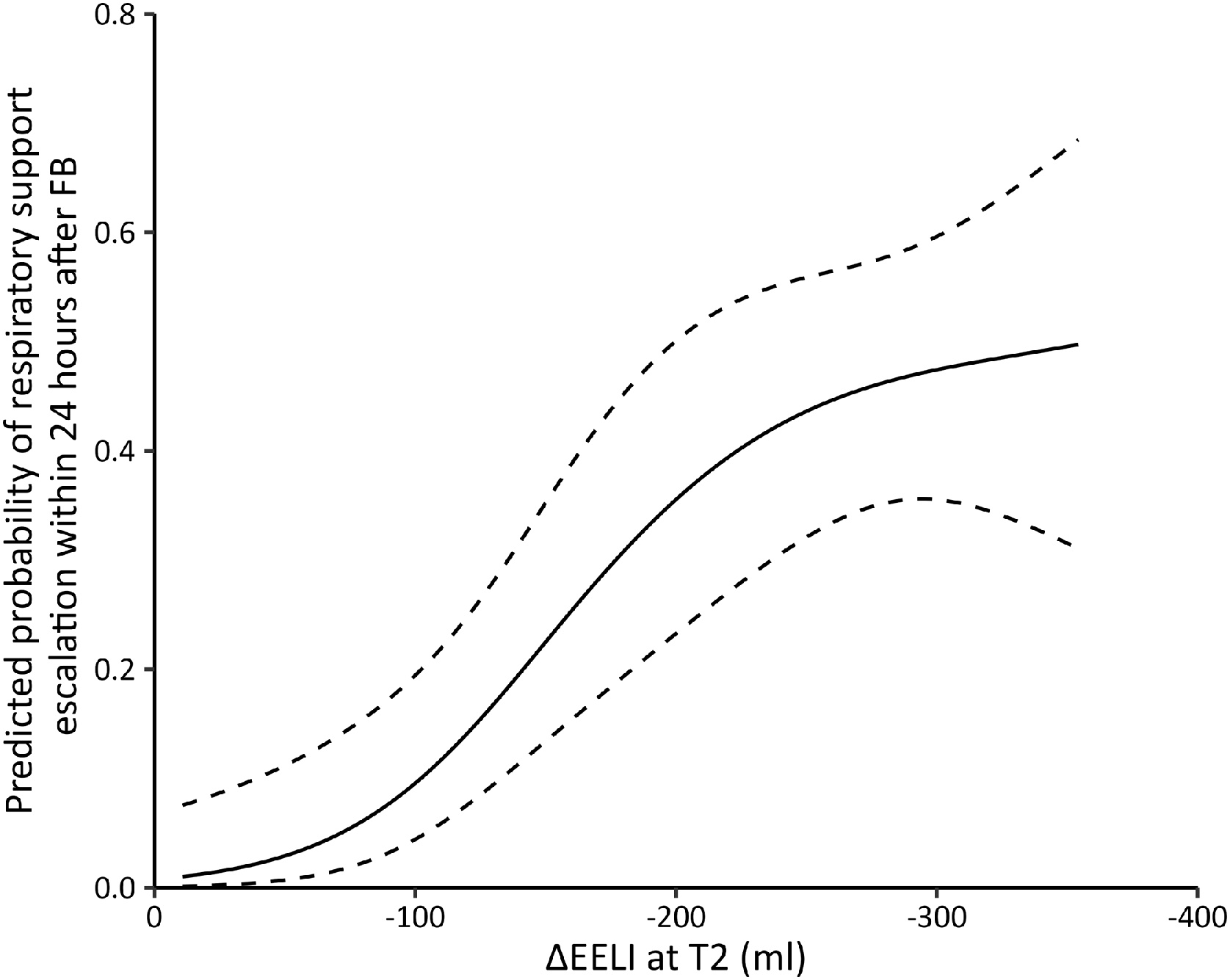
Predicted probability of respiratory support escalation within 24 h after FB based on ΔEELI measured at T2. FB flexible bronchoscopy, ΔEELI the changes in end-expiratory lung impedance.

Figure S4 shows representative EIT images from both groups. Differences in EIT measurements were observed between the two groups (Table 3 and Fig. 4). TIV decreased during FB in both groups but was better sustained in the HFNO-SPC group at T2 (*P* = 0.024), suggesting more stable ventilation during the procedure. ΔEELI decreased significantly during FB in both groups. However, the magnitude of reduction was consistently smaller in the HFNO-SPC group at T2, T3, and T4 (all *P* < 0.01), indicating better maintenance of EELV over time. ΔEELI at T2 was modeled using restricted cubic splines to account for potential non-linearity. The resulting curve exhibited a biphasic pattern, with the predicted probability of respiratory support escalation within 24 h after FB increasing more steeply when ΔEELI exceeded approximately 100 ml (Fig. 5). Similar spline transformations were also applied to ΔEELI at T3 and T4 (Figure S5 and Figure S6).

Before FB, arterial blood gas parameters did not differ significantly between groups. At T4, the HFNO-SPC group exhibited significantly higher PaO_2_ (79.7 ± 16.3 mmHg vs. 74.4 ± 12.5 mmHg, *P* = 0.022) and PaO_2_/FiO_2_ (171.1 [IQR, 147.2–190.0] mmHg vs. 158.6 [IQR, 134.1–182.1] mmHg, *P* = 0.013) compared with the SOT group (Table S5 and Figure S7).

### Bronchoscopic procedures and related events

Bronchoscopic procedures were similar between groups (Table 4). Most patients (95.6%) underwent BAL, with no significant differences in the amount of fluid instilled or recovered. The proportions of patients receiving bronchial brushing, endobronchial biopsy, or transbronchial lung biopsy were also comparable between groups. Adverse events during and within 24 h after FB occurred at similar rates in both groups, with no statistically significant differences observed.

**Table 4:** Bronchoscopic procedure and related events.

Variables	HFNO-SPC group (n = 80)	SOT group (n = 80)	*P*
Bronchoscopic procedure			
Bronchoalveolar lavage, no. (%)	78 (97.5)	75 (93.8)	0.443
Amount of fluid instilled, ml	60 (40–80)	60 (45–70)	0.978
Amount of fluid recovered, ml	30 (24–40)	31 (20–40)	0.947
Bronchial brushing, no. (%)	60 (75.0)	51 (63.8)	0.123
Endobronchial biopsy, no. (%)	28 (35.0)	30 (37.5)	0.742
Transbronchial lung biopsy, no. (%)	15 (18.8)	16 (20.0)	0.841
Events during FB, no. (%)			
Agitation	18 (22.5)	24 (30.0)	0.281
Bronchospasm	2 (2.5)	5 (6.3)	0.443
Arrhythmias or tachycardia ≥150 beats/min	7 (8.8)	8 (10.0)	0.786
Hypertension (systolic BP >180 mmHg)	11 (13.8)	14 (17.5)	0.514
Epistaxis	4 (5.0)	4 (5.0)	1.000
Mucosal bleeding	18 (22.5)	21 (26.3)	0.581
Events within 24 h post-FB, no. (%)			
Transient fever	21 (26.3)	17 (21.3)	0.457
Pneumothorax	2 (2.5)	3 (3.8)	1.000
Hemorrhage	4 (5.0)	6 (7.5)	0.514

HFNO-SPC high-flow nasal oxygen therapy via a single-prong cannula interface, SOT standard oxygen therapy, FB flexible bronchoscopy, BP blood pressure.

## Discussion

This is the first RCT to assess clinical outcomes following FB in patients with ARF, demonstrating that HFNO-SPC significantly lowered the incidence of respiratory support escalation in the first 24 h after FB compared with SOT. Secondary analysis using a hierarchical win ratio approach further supported this benefit across clinically ranked escalation events. Patients in the HFNO-SPC group also exhibited greater stability of oxygenation and ventilation, as evidenced by higher PaO_2_/FiO_2_ after FB and smaller reductions in TIV and EELI during and after the procedure.

FB could compromise respiratory mechanics and exacerbate respiratory failure, especially in patients with underlying lung disease. The bronchoscopes used in this study had outer diameters of 4.9 mm and 5.9 mm, occupying approximately 10% and 15% of the tracheal cross-sectional area, respectively. Its insertion caused partial airway obstruction, increased the respiratory load, and contributed to hypoxemia [18]. The high flow rates delivered by HFNO-SPC reduce airway resistance and allow for better maintenance of tidal volume during FB compared with SOT [19]. Suctioning during FB generates negative pressure, and the instillation of local anesthetics or saline for BAL can cause alveolar collapse [20]. These effects reduce EELV and impair ventilation-perfusion (V/Q) matching, ultimately leading to more profound hypoxemia [21]. Under ideal conditions, HFNO therapy delivered at 60 L/min with the mouth closed can generate approximately 7 cmH_2_O of expiratory positive airway pressure [13]. This pressure provides alveolar support and may reduce the decline in EELI during and after FB [22]. Consistent with previous findings, this mechanism may explain the better preservation of oxygenation observed in the HFNO-SPC group during FB [15].

A recent meta-analysis has demonstrated that HFNO reduces desaturation and procedure-related interruptions during bronchoscopy [23]; however, this study primarily focused on physiological endpoints rather than clinically meaningful outcomes. Consistent with these findings, prior studies including one conducted at our center have demonstrated that HFNO is more effective in preserving SpO_2_ than nasal cannula or facemask oxygen during bronchoscopy [12,16,[24], [25], [26]]. However, we believe that the need for respiratory support escalation following FB may carry greater clinical relevance than transient desaturation occurring during the procedure. Our study demonstrated that a significantly higher proportion of patients in the SOT group required respiratory support escalation within 24 h after FB compared with the HFNO-SPC group (33.8% vs. 15.0%, *P* = 0.006). Notably, the need for endotracheal intubation, reflecting a significant deterioration in respiratory status, was also more frequent in the SOT group (20.0% vs. 7.5%, *P* = 0.022). As both groups received individualized noninvasive respiratory strategies before and after FB, the difference is likely attributable to the type of respiratory support used during the procedure. Notably, this increased need for escalation did not translate into significant differences in mortality between the groups.

However, some studies suggest that respiratory deterioration after FB is more likely to reflect the natural course of the underlying disease rather than the direct impact of the procedure itself [2,5,6,27]. Consistent with this perspective, longer-term outcomes in our study, including intubation at 7 and 28 days and mortality, did not differ significantly between groups, suggesting that these endpoints were primarily driven by the underlying disease process. In contrast, the significantly higher rates of respiratory support escalation, including intubation, observed within 24 h after FB in the SOT group indicate that early deterioration was more closely related to the type of respiratory support used during the procedure.

In our study, patients in the SOT group exhibited a more pronounced decline in EELI during FB, followed by a slower recovery thereafter. EIT monitoring showed that a ΔEELI exceeding 100 mL at T2 and 75 mL at T4 was significantly associated with an increased risk of respiratory support escalation. Following FB, patients in the SOT group likely required greater transpulmonary and transdiaphragmatic pressures to facilitate alveolar recruitment, indicating a high respiratory drive [28,29]. Sustained high respiratory drive can result in strong inspiratory efforts, leading to patient-ventilator asynchrony and elevated lung-distending pressures, both of which substantially contribute to the development of ventilator-induced lung injury (VILI) and patient self-inflicted lung injury (P-SILI) [30,31]. Prolonged excessive respiratory effort may eventually lead to respiratory muscle fatigue, thereby increasing the need for escalation to NIV or IMV. At T4, the partial pressure of arterial carbon dioxide (PaCO_2_) was tended to be lower in the SOT group compared to the HFNO-SPC group (34.7 ± 8.4 mmHg vs. 37.1 ± 8.7 mmHg, *P* = 0.079). This reduction in the SOT group may indicate the development of a rapid, shallow breathing pattern secondary to inspiratory muscle fatigue [32].

HFNO-SPC was marked by smoother fluctuations in vital signs throughout FB, which may partly explain its association with a lower need for respiratory support escalation. However, these differences in vital signs were modest and transient, and may not fully account for the observed differences in clinical outcomes. These physiological fluctuations observed in patients undergoing FB were likely attributable to stress-induced sympathetic overactivity. Compared to SOT, the HFNO-SPC provided greater comfort and more stable oxygen delivery, which may have alleviated patient discomfort and anxiety thereby contributing to the lower respiratory rate, heart rate, and mean arterial pressure observed in the patients receiving HFNO-SPC [33,34]. In addition, patients in the HFNO-SPC group exhibited better recovery of oxygenation after FB, as reflected by higher PaO_2_ and PaO_2_/FiO_2_ levels at 2 h after the procedure. More importantly, EIT-derived parameters, particularly ΔEELI, showed significant and sustained differences between groups during and after FB, suggesting more pronounced lung derecruitment in the SOT group.

Previous studies have reported that NIV is more effective than HFNO in improving oxygenation during FB [5,6]. In those studies, the procedure was performed via the oral route with a bite block, requiring the mouth to remain open. This setup may lead to gas leakage, impairing both the generation and maintenance of positive expiratory airway pressure and thereby increasing the risk of FB-related hypoxemia [13]. Although NIV reduces the risk of hypoxemia during FB, its limited tolerance may accelerate respiratory deterioration and increase the risk of intubation [5,6]. Moreover, inserting and maneuvering the bronchoscope through an orifice of the NIV interface can be technically challenging, requiring experienced bronchoscopists and time for their adaptation [35]. In line with these concerns, one study reported that among 49 non-intubated patients with severe respiratory failure, only 8.2% (4/49) underwent FB with NIV support [3]. HFNO is increasingly adopted as an alternative to NIV for patients with ARF because it is easy to use and better tolerated. The single-prong cannula of the HFNO-SPC interface leaves one nostril free for bronchoscope insertion, enabling better control of the bronchoscope [36]. The absence of a bite block allows patients to keep their mouths closed, which may help maintain positive expiratory airway pressure during FB. To ensure that the observed differences were attributable to the support modality rather than to differences in FiO_2_, the control group received oxygen via a non-rebreathing reservoir mask capable of delivering an FiO_2_ of up to 0.8, comparable to HFNO-SPC.

Beyond being the first RCT to evaluate post-FB outcomes with HFNO-SPC in patients with ARF, our study has several limitations that should be acknowledged. First, this was an open-label study because the HFNO device and non-rebreathing mask were visibly different. Although patients were not explicitly informed of group allocation and most outcomes were objective and predefined, the possibility of clinical decision-making bias could not be entirely excluded. Second, although escalation criteria were predefined, clinician judgment in borderline cases might still have been influenced by awareness of group allocation. Third, post-FB noninvasive respiratory support was not governed by a mandatory protocol. In general, patients resumed their pre-FB respiratory support after the procedure, with escalation undertaken as needed according to the predefined respiratory support escalation strategy. Fourth, although the EIT belt position was marked to minimize displacement and EELI was expressed relative to TIV to enhance comparability, the values obtained at T4 may still be influenced by changes in patient position or signal drift over time [37]. Fifth, transcutaneous carbon dioxide (PtCO_2_) was not monitored during FB, which limited our ability to assess differences in CO_2_ clearance between HFNO-SPC and SOT group. Although arterial blood gases were obtained at T0 and T4, dynamic changes in PaCO_2_ levels throughout FB were not monitored. Last, esophageal and gastric balloon catheters were not placed due to poor tolerance in most awake patients. Therefore, transpulmonary and transdiaphragmatic pressures were not monitored and were assessed qualitatively based on breathing patterns, which may not accurately reflect respiratory effort.

## Conclusion

In patients with ARF, HFNO-SPC significantly reduced the need for respiratory support escalation within 24 h after FB compared with SOT. This benefit was consistently observed across a hierarchically ordered composite outcome reflecting escalating levels of respiratory support. These findings support HFNO-SPC as a clinically effective and physiologically advantageous oxygenation strategy during FB. However, the applicability of this intervention to routine clinical practice warrants further evaluation.

## Author contributions

RW contributed substantially to the study conception and design, supervised and coordinated the study implementation, recruited patients, participated in data acquisition and analysis, drafted the initial manuscript, and revised the manuscript based on co-author feedback. YZ and WCW contributed to the acquisition of clinical data and provided critical revisions to the manuscript. NL was responsible for statistical analysis and interpretation of the data. XT, TL, XQL, HCL, ML, ZX, and LW participated in patient care and data collection. BS and GG were actively involved in monitoring and organizing the study and contributed to patient recruitment. All authors reviewed and approved the final version of the manuscript.

## Consent for publication

Consent for publication was obtained for this report.

## Ethics approval and consent to participate

This study is approved by the ethics committee of Beijing Chao-Yang Hospital and Qinghai University Affiliated Hospital.

## Funding

The Beijing Municipal Administration of Hospitals Incubating Program (PX2023010).

## Availability of data and materials

The data are available from the corresponding author on reasonable request.

## Declaration of competing interest

The authors declare that there are no financial or personal relationships that could inappropriately influence (bias) the work reported in this manuscript.
