# Supplementary material for: High-flow nasal oxygen therapy via a single-prong cannula interface during bronchoscopy in patients with acute respiratory failure: a two-center, open-label, randomized controlled trial
Source: Ann Intensive Care. 2026 May 12;16:100081. doi: 10.1016/j.aicoj.2026.100081 (PMC13195340; doi:10.1016/j.aicoj.2026.100081)
Supplement: Supplementary file 1 [file mmc1.docx]

**ADDITIONAL FILE 1: SUPPLEMENTARY MATERIAL**

**High-Flow Nasal Oxygen Therapy via a Single-Prong Cannula Interface During Bronchoscopy in Patients with Acute Respiratory Failure: A Two-Center, Open-Label, Randomized Controlled Trial**

**Corresponding author:**

Rui Wang MD

Department of Respiratory and Critical Care Medicine, Beijing Institute of Respiratory Medicine and Beijing Chao-Yang Hospital, Capital Medical University, No. 8 Gongren Tiyuchang Nanlu, Chaoyang District, Beijing, China.

Email: wangrui1985816@gmail.com

**1. Online Methods Supplement**

**1.1 Section S1 Calibration and computation of** **electrical impedance tomography-derived tidal volume and changes in end-expiratory lung impedance**

Electrical impedance tomography (EIT) recordings were processed offline by selecting five consecutive respiratory cycles from visually stable segments at each predefined time point (T0-T4). The following steps were undertaken:

1. Calibration at baseline (T0)

(1) Patients were ensured to be in a stable condition with minimal leak (<20 L/min), allowing reliable tidal volume (TV) measurement via the NIV device (Philips Respironics V60; Philips Respironics, Murrysville, PA, USA). NIV was delivered using a Mirage Quattro™ oronasal mask (ResMed, San Diego, CA, USA).

(2) For the same cycles, tidal impedance variation (TIV) was computed as the difference between end-inspiratory and end-expiratory impedance.

(3) A calibration factor (*k*) was derived as:

$$k=\frac{{TV}_{T0}}{{TIV}_{T0}}$$

2. Estimation of tidal volume (T1-T4)

At each subsequent time point, TIV was calculated in the same way. Estimated tidal volume was obtained as:

$${TV}_{Ti}=k\times{TIV}_{Ti} \left( i=1, 2, 3, 4 \right)$$

3. Assessment of lung volume changes

(1) End-expiratory lung impedance (EELI) was measured at each time point.

(2) The relative change in end-expiratory impedance (ΔEELI) compared with baseline was calculated as:

$${\Delta EELI}_{Ti-T0}={EELI}_{Ti}-{EELI}_{T0}$$

(3) Corresponding changes in end-expiratory lung volume (ΔEELV) were estimated as:

$${\Delta EELV}_{Ti-T0}={\Delta EELI}_{Ti-T0}\times k$$

**1.2 Section S2 Flexible bronchoscopy-associated adverse events**

Adverse events associated with flexible bronchoscopy (FB) included agitation, bronchospasm, arrhythmias, tachycardia (≥150 beats/min), hypertension (systolic blood pressure >180 mmHg), epistaxis, and mucosal bleeding. Four main types of arrhythmias were recorded: premature beats, supraventricular tachycardia, ventricular arrhythmias, and bradyarrhythmia.

Adverse events within 24 hours of FB included transient fever (temperature ≥38.5 °C), pneumothorax, and hemorrhage. Transient fever, defined as a self-limited temperature elevation resolving within 24 hours, was the most commonly observed post-FB event, particularly following bronchoalveolar lavage (BAL). In patients undergoing transbronchial lung biopsy, routine post-FB chest radiography was performed to screen for pneumothorax. Clinically significant pneumothorax was managed promptly with chest tube insertion to prevent oxygen desaturation or progression to tension pneumothorax. Hemorrhagic events were managed with topical instillation of diluted epinephrine or, when necessary, more advanced bronchoscopic interventions [1].

**1.3 Section S3 Respiratory support escalation strategy**

For patients receiving low-flow nasal oxygen (LFNO) who failed to maintain adequate oxygenation, stepwise escalation to high-flow nasal oxygen (HFNO) and subsequently to NIV was permitted. For those already receiving HFNO, a trial of NIV was allowed at the physician’s discretion in cases of persistent or worsening respiratory failure, provided no other organ dysfunction was present prior to proceeding to endotracheal intubation and IMV [2].

Endotracheal intubation and initiation of invasive mechanical ventilation (IMV) were considered in patients with:

1. Signs of persistent or worsening respiratory failure, defined by at least two of the following:

(1) Respiratory rate > 40 breaths/min;

(2) No improvement in respiratory muscle fatigue;

(3) Copious tracheal secretions;

(4) Acidosis with a pH < 7.35;

(5) SpO_2_ < 90% for more than 5 minutes;

(6) Intolerance to noninvasive ventilation (NIV).

2. Hemodynamic instability, defined by any of the following:

(1) Systolic blood pressure < 90 mmHg;

(2) Mean arterial pressure < 65 mmHg;

(3) Requirement for vasopressors.

3. Neurologic deterioration, defined as a Glasgow Coma Scale score < 12.

**2. Online Tables Supplement**

2.1 Table S1. Baseline laboratory tests at randomization

| **Variables** | **All patients (n = 160)** | **HFNO-SPC group (n = 80)** | **SOT group (n = 80)** | ***P*** |
| --- | --- | --- | --- | --- |
| White blood cell, ×10^9^/L | 10.11 ± 3.84 | 10.05 ± 4.19 | 10.17 ± 3.49 | 0.836 |
| Neutrophil, ×10^9^/L | 8.30 ± 3.59 | 8.11 ± 3.37 | 8.48 ± 3.80 | 0.517 |
| Lymphocyte, ×10^9^/L | 0.89 ± 0.60 | 0.86 ± 0.58 | 0.93 ± 0.62 | 0.496 |
| Hemoglobin, g/L | 110 ± 24 | 109 ± 24 | 111 ± 24 | 0.697 |
| Platelet, ×10^9^/L | 212 (157 to 271) | 212 (152 to 267) | 215 (161 to 282) | 0.688 |
| Albumin, g/L | 32.6 (30.1 to 36.2) | 32.6 (30.6 to 36.5) | 32.6 (29.7 to 36.0) | 0.600 |
| Aspartate aminotransferase, U/L | 38 (17 to 71) | 36 (17 to 69) | 42 (22 to 74) | 0.259 |
| Alanine aminotransferase, U/L | 41 (13 to 85) | 38 (12 to 89) | 45 (21 to 76) | 0.605 |
| Total bilirubin, μmol/L | 10.4 (7.7 to 14.9) | 10.5 (7.5 to 16.0) | 10.2 (7.8 to 14.2) | 0.702 |
| Direct bilirubin, μmol/L | 6.1 (4.6 to 7.9) | 6.2 (4.1 to 8.4) | 6.1 (5.1 to 7.6) | 0.623 |
| Blood urea nitrogen, mmol/L | 7.79 (4.71 to 11.07) | 7.27 (4.40 to 10.67) | 7.93 (5.19 to 11.87) | 0.343 |
| Creatinine, μmol/L | 68.5 (48.8 to 93.9) | 68.4 (46.4 to 90.0) | 70.9 (50.2 to 107.1) | 0.316 |
| Sodium, mmol/L | 138.3 ± 3.8 | 138.6 ± 3.8 | 138.0 ± 3.8 | 0.366 |
| Potassium, mmol/L | 4.0 ± 0.5 | 4.0 ± 0.5 | 4.0 ± 0.4 | 0.760 |
| Glucose, mmol/L | 6.85 (5.29 to 8.40) | 6.34 (5.20 to 8.35) | 7.15 (5.40 to 8.57) | 0.184 |
| Prothrombin time, s | 12.3 (11.3 to 13.3) | 12.4 (11.5 to 13.4) | 12.3 (11.2 to 13.2) | 0.657 |
| Activated partial thromboplastin time, s | 29.3 (26.3 to 34.7) | 28.8 (26.3 to 33.5) | 30.1 (26.3 to 36.4) | 0.301 |
| Fibrinogen, mg/dl | 430.8 ± 167.1 | 432.6 ± 164.9 | 429.0 ± 170.3 | 0.894 |
| D-Dimer, mg/L | 1.50 (0.79 to 2.99) | 1.51 (0.77 to 3.13) | 1.46 (0.83 to 2.82) | 0.912 |
| N-terminal pro B-type natriuretic peptide, pg/ml | 559 (239 to 1638) | 522 (230 to 1875) | 650 (268 to 1565) | 0.737 |
| C-reaction protein, mg/L | 62.3 (25.6 to 95.8) | 63.0 (28.1 to 98.5) | 58.7 (20.8 to 91.9) | 0.364 |
| Procalcitonin, ng/ml | 0.22 (0.08 to 0.69) | 0.16 (0.07 to 0.62) | 0.26 (0.10 to 1.06) | 0.165 |

HFNO-SPC high-flow nasal oxygen therapy via a single-prong cannula interface, SOT standard oxygen therapy

2.2 Table S2. Respiratory support escalation within 24h after FB in the HFNO-SPC and SOT groups

|  | **All patients (n = 160)** | **HFNO-SPC group (n = 80)** | **SOT group (n = 80)** | ***P*** |
| --- | --- | --- | --- | --- |
| Maintained the same respiratory support level | 121 (75.6) | 68 (85.0) | 53 (66.3) | 0.006 |
| LFNO, then HFNO therapy | 3 (1.9) | 2 (2.5) | 1 (1.3) | 1.000 |
| LFNO, then HFNO therapy, then NIV | 1 (0.6) | 0 (0.0) | 1 (1.3) | 1.000 |
| HFNO therapy, then flow rate or FiO_2_ increase > 20% | 4 (2.5) | 1 (1.3) | 3 (3.8) | 0.620 |
| HFNO therapy, then NIV | 7 (4.4) | 2 (2.5) | 5 (6.3) | 0.443 |
| HFNO therapy, then NIV, then IMV | 14 (8.8) | 3 (3.8) | 11 (13.8) | 0.025 |
| NIV, then IPAP, EPAP, or FiO_2_ increase > 20% | 2 (1.3) | 1 (1.3) | 1 (1.3) | 1.000 |
| NIV, then IMV | 8 (5.0) | 3 (3.8) | 5 (6.3) | 0.719 |

FB flexible bronchoscopy, HFNO-SPC high-flow nasal oxygen therapy via a single-prong cannula interface, SOT standard oxygen therapy, LFNO low-flow nasal oxygen, NIV noninvasive ventilation, FiO_2_ fraction of inspired oxygen, IMV invasive mechanical ventilation, IPAP inspiratory positive airway pressure, EPAP expiratory positive airway pressure

2.3 Table S3. Clinical outcomes in the per-protocol analysis

| **Outcomes** | **HFNO-SPC group**  **(n = 77)** | **SOT group**  **(n = 76)** | **Mean, median, or risk difference, (95% CI)** | **Relative difference, (95% CI)** | ***P*** |
| --- | --- | --- | --- | --- | --- |
| **Primary outcome** |  |  |  |  |  |
| Respiratory support escalation within 24h post-FB, no. (%) | 12 (15.6) | 26 (34.2) | -18.6 (-31.9 to - 5.0)^a^ | HR, 0.406 (0.205 to 0.805) | 0.008^b^ |
| **Secondary outcome** |  |  |  |  |  |
| Respiratory support escalation within 24h post-FB tested in prespecified fixed sequence, no. (%) | 1866 (31.8) | 733 (12.5) | 19.4 (4.6 to 34.1) | WR, 2.546 (1.311 to 4.943) | 0.006^c^ |
| **Other outcomes** |  |  |  |  |  |
| Intubation within 24h post-FB, no. (%) | 6 (7.8) | 15 (19.7) | -11.9 (-23.4 to -1.1)^a^ | HR, 0.368 (0.143 to 0.949) | 0.032^b^ |
| Intubation within 7d post-FB, no. (%) | 16 (20.8) | 24 (31.6) | -10.8 (-24.6 to 3.0)^a^ | HR, 0.611 (0.324 to 1.150) | 0.129^b^ |
| Intubation within 28d post-FB, no. (%) | 19 (24.7) | 26 (34.2) | -9.5 (-23.9 to 4.8)^a^ | HR, 0.664 (0.367 to 1.199) | 0.196^b^ |
| Lowest SpO_2_ during FB, % | 90 (88 to 92) | 86 (83 to 88) | 4.0 (3.0 to 5.5)^d^ | N/A^e^ | < 0.001^f^ |
| Number of patients with interrupted FB, no. (%) | 36 (46.8) | 48 (63.2) | -16.4 (-32.0 to -0.9)^g^ | OR, 0.512 (0.266 to 0.973) | 0.041^b^ |
| Duration of FB, min | 10.7 ± 4.3 | 12.2 ± 4.7 | -1.581 (-3.204 to -0.138) | HR, 1.371 (0.995 to 1.890) | 0.032^h^ |
| ICU length of stay, days | 13 (10 to 20) | 17 (11 to 23) | -3.500 (-6.000 to 0.500)^d^ | HR, 1.121 (0.815 to 1.542) | 0.215^f^ |
| Hospital length of stay, days | 19 (15 to 24) | 20 (17 to 26) | -0.500 (-4.000 to 2.500)^d^ | HR, 1.061 (0.769 to 1.462) | 0.404^f^ |
| 28-day mortality, no. (%) | 11 (14.3) | 16(21.1) | -6.8 (-19.1 to 5.5)^a^ | HR, 0.667 (0.309 to 1.436) | 0.272^b^ |
| 60-day mortality, no. (%) | 15 (19.5) | 20 (26.3) | -6.8 (-20.2 to 6.6)^a^ | HR, 0.713 (0.365 to 1.393) | 0.314^b^ |
| 90-day mortality, no. (%) | 17 (22.1) | 21 (27.6) | -5.6 (-19.2 to 8.1)^g^ | HR, 0.766 (0.404 to 1.453) | 0.427^b^ |

HFNO-SPC high-flow nasal oxygen therapy via a single-prong cannula interface, SOT standard oxygen therapy, FB flexible bronchoscopy, SpO_2_ peripheral oxygen saturation, ICU intensive care unit, HR, hazard ratio, WR win ratio, N/A not applicable, OR odds ratio

^a^ The 95% confidence interval for the rate difference was estimated using the Miettinen-Nurminen method.

^b^ *P* values were derived from chi-square tests.

^c^ Secondary outcomes defined per statistical analysis plan.

^d^ The 95% confidence interval for the median difference was estimated based on 5000 bootstrap samples.

^e^ This measure is not applicable to continuous outcomes.

^f^ *P* values were derived from Mann-Whitney U tests.

^g^ The 95% confidence interval for the rate difference was estimated using the Wald method.

^h^ *P* values were derived from t tests.

2.4 Table S4. Clinical characteristics of patients intubated within 24 hours after FB

| **Patients** | **Age, years** | **APACHE II score** | **Diagnosis** | **Respiratory support pre-FB** | **PaO_2_/FiO_2_ at randomization, mm Hg** | **Duration of FB, min** | **ΔEELI at T2, ml** | **Time to intubation post-FB, h** | **28-day survival status** |
| --- | --- | --- | --- | --- | --- | --- | --- | --- | --- |
| **HFNO-SPC group** |  |  |  |  |  |  |  |  |  |
| 1 | 65 | 22 | Severe CAP | NIV | 160 | 13.3 | 279 | 10 | Died |
| 2 | 75 | 9 | HAP | NIV | 152 | 11.7 | 243 | 7 | Died |
| 3 | 63 | 20 | Severe CAP | HFNO | 194 | 12.8 | 204 | 21 | Survived |
| 4 | 74 | 15 | ILD | HFNO | 155 | 14.0 | 264 | 14 | Died |
| 5 | 73 | 18 | PJP | HFNO | 161 | 11.0 | 226 | 3 | Died |
| 6 | 77 | 21 | Severe CAP | NIV | 150 | 7.5 | 224 | 13 | Survived |
| **SOT group** |  |  |  |  |  |  |  |  |  |
| 1 | 78 | 21 | HAP | HFNO | 175 | 12.1 | 269 | 4 | Died |
| 2 | 71 | 19 | Severe CAP | HFNO | 158 | 15.5 | 384 | 3 | Died |
| 3 | 63 | 25 | PJP | NIV | 237 | 9.3 | 307 | 5 | Survived |
| 4 | 75 | 9 | Lung adenocarcinoma | HFNO | 162 | 8.4 | 354 | 8 | Died |
| 5 | 62 | 7 | Severe CAP | HFNO | 156 | 6.0 | 81 | 6 | Died |
| 6 | 66 | 5 | ILD | NIV | 150 | 13.3 | 146 | 4 | Died |
| 7 | 66 | 12 | Severe CAP | HFNO | 192 | 9.9 | 296 | 6 | Died |
| 8 | 69 | 14 | ILD | HFNO | 178 | 15.3 | 352 | 16 | Died |
| 9 | 86 | 23 | HAP | HFNO | 236 | 10.3 | 221 | 8 | Survived |
| 10 | 62 | 10 | Severe CAP | NIV | 152 | 11.2 | 348 | 2 | Died |
| 11 | 58 | 8 | PJP | HFNO | 183 | 10.3 | 260 | 8 | Survived |
| 12 | 66 | 18 | Severe CAP | NIV | 160 | 8.4 | 318 | 22 | Died |
| 13 | 75 | 16 | ILD | HFNO | 157 | 14.0 | 163 | 16 | Survived |
| 14 | 39 | 15 | MPA | HFNO | 150 | 7.4 | 320 | 10 | Survived |
| 15 | 78 | 17 | Severe CAP | HFNO | 187 | 7.3 | 334 | 14 | Died |
| 16 | 46 | 20 | Severe CAP | NIV | 153 | 12.2 | 314 | 20 | Survived |

FB flexible bronchoscopy, APACHE II Acute Physiology and Chronic Health Evaluation II, PaO_2_/FiO_2_ the ratio of the partial pressure of arterial oxygen to the fraction of inspired oxygen, ΔEELI the changes in end-expiratory lung impedance, HFNO-SPC high-flow nasal oxygen therapy via a single-prong cannula interface, CAP community-acquired pneumonia, NIV noninvasive ventilation, HAP hospital-acquired pneumonia, ILD interstitial lung disease, PJP pneumocystis jirovecii pneumonia, SOT standard oxygen therapy, MPA microscopic polyangiitis

2.5 Table S5. Comparison of arterial blood gas parameters between the HFNO-SPC and SOT groups at T0 and T4

| **Variables** | **HFNO-SPC group (n = 80)** | **SOT group (n = 80)** | ***P*** |
| --- | --- | --- | --- |
| **Arterial blood gas at T0** |  |  |  |
| pH | 7.45 (7.40 to 7.49) | 7.45 (7.40 to 7.50) | 0.444 |
| PaO_2_, mmHg | 78.5 ± 10.9 | 80.6 ± 11.5 | 0.240 |
| PaCO_2_, mmHg | 37.4 ± 8.5 | 37.5 ± 7.4 | 0.929 |
| HCO_3_^-^, mmol/L | 26.3 ± 4.3 | 25.8 ± 4.1 | 0.466 |
| PaO_2_/FiO_2_, mmHg | 181.0 (160.8 to 205.9) | 186.3 (164.2 to 209.8) | 0.573 |
| **Arterial blood gas at T4** |  |  |  |
| pH | 7.46 (7.43 to 7.48) | 7.46 (7.42 to 7.49) | 0.538 |
| PaO_2_, mmHg | 79.7 ± 16.3 | 74.4 ± 12.5 | 0.022 |
| PaCO_2_, mmHg | 37.1 ± 8.7 | 34.7 ± 8.4 | 0.079 |
| HCO_3_^-^, mmol/L | 26.2 ± 3.8 | 25.9 ± 3.4 | 0.700 |
| PaO_2_/FiO_2_, mmHg | 171.1 (147.2 to 190.0) | 158.6 (134.1 to 182.1) | 0.013 |

HFNO-SPC high-flow nasal oxygen therapy via a single-prong cannula interface, SOT standard oxygen therapy, PaO_2_ partial pressure of arterial oxygen, PaCO_2_ partial pressure of arterial carbon dioxide, HCO_3_^-^ bicarbonate, PaO_2_/FiO_2_ the ratio of the partial pressure of arterial oxygen to the fraction of inspired oxygen

**3. Online Figures Supplement**

3.1 Figure S1 Win ratio diagram for the secondary outcome in the intention-to-treat analysis.


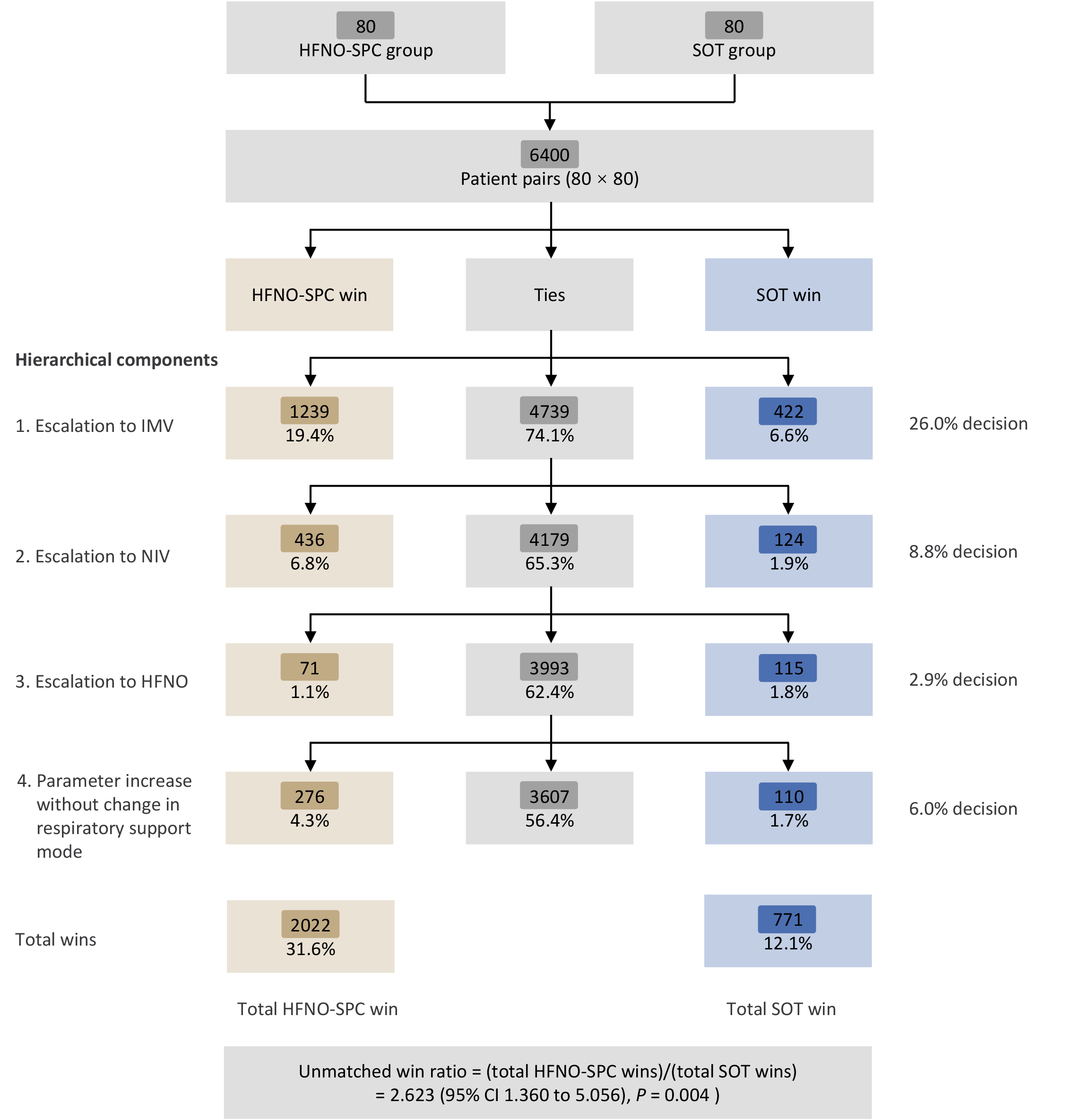


HFNO-SPC high-flow nasal oxygen therapy via a single-prong cannula interface, SOT standard oxygen therapy, IMV invasive mechanical ventilation, NIV noninvasive ventilation, HFNO high-flow nasal oxygen

3.2 Figure S2 Win ratio diagram for the secondary outcome in the per-protocol analysis.


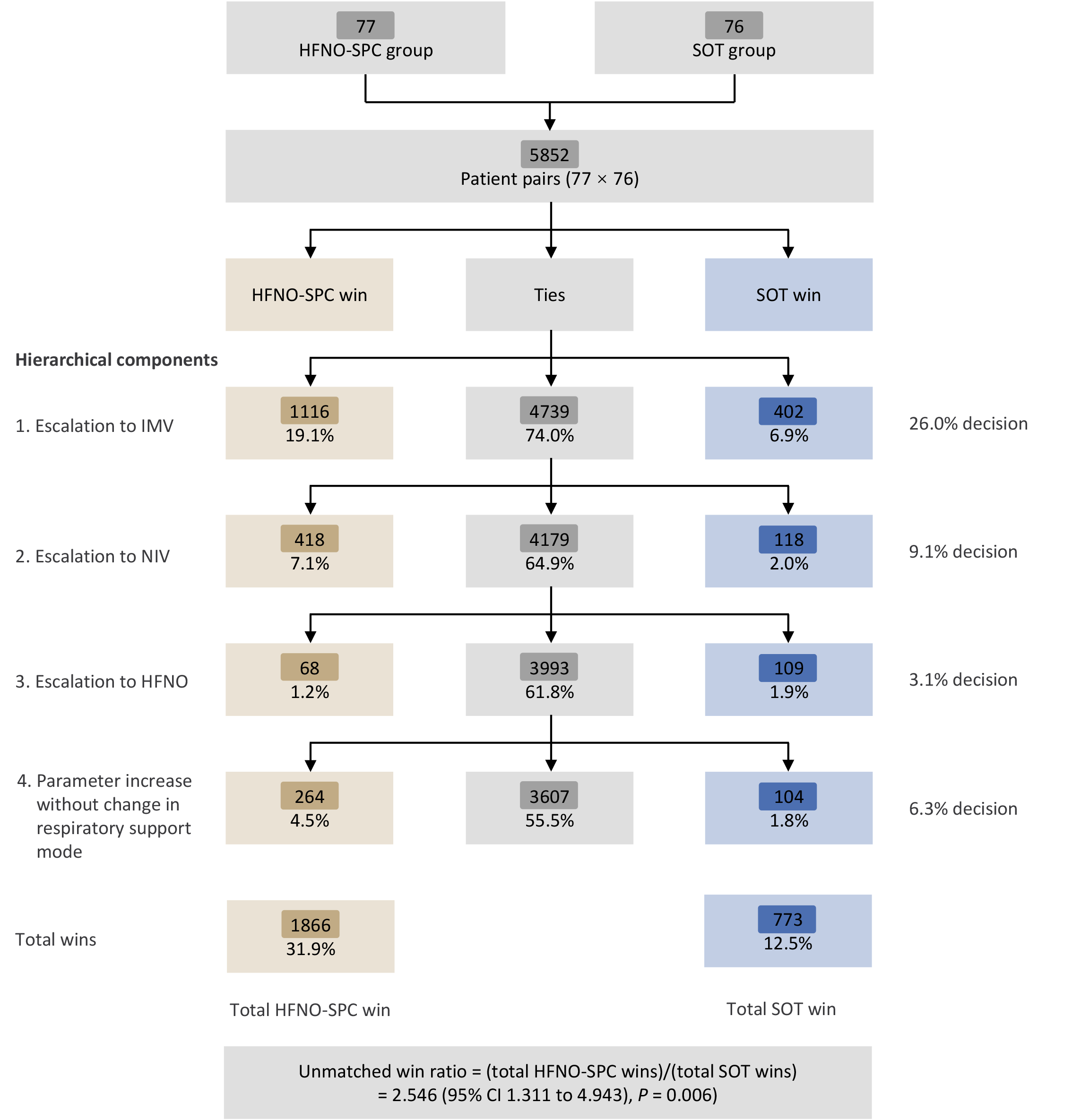


HFNO-SPC high-flow nasal oxygen therapy via a single-prong cannula interface, SOT standard oxygen therapy, IMV invasive mechanical ventilation, NIV noninvasive ventilation, HFNO high-flow nasal oxygen

3.3 Figure S3 Comparison of peripheral oxygen saturation **(A)**, respiratory rate **(B)**, heart rate **(C)**, and mean arterial pressure **(D)** between the HFNO-SPC group and the SOT group at predefined time points.


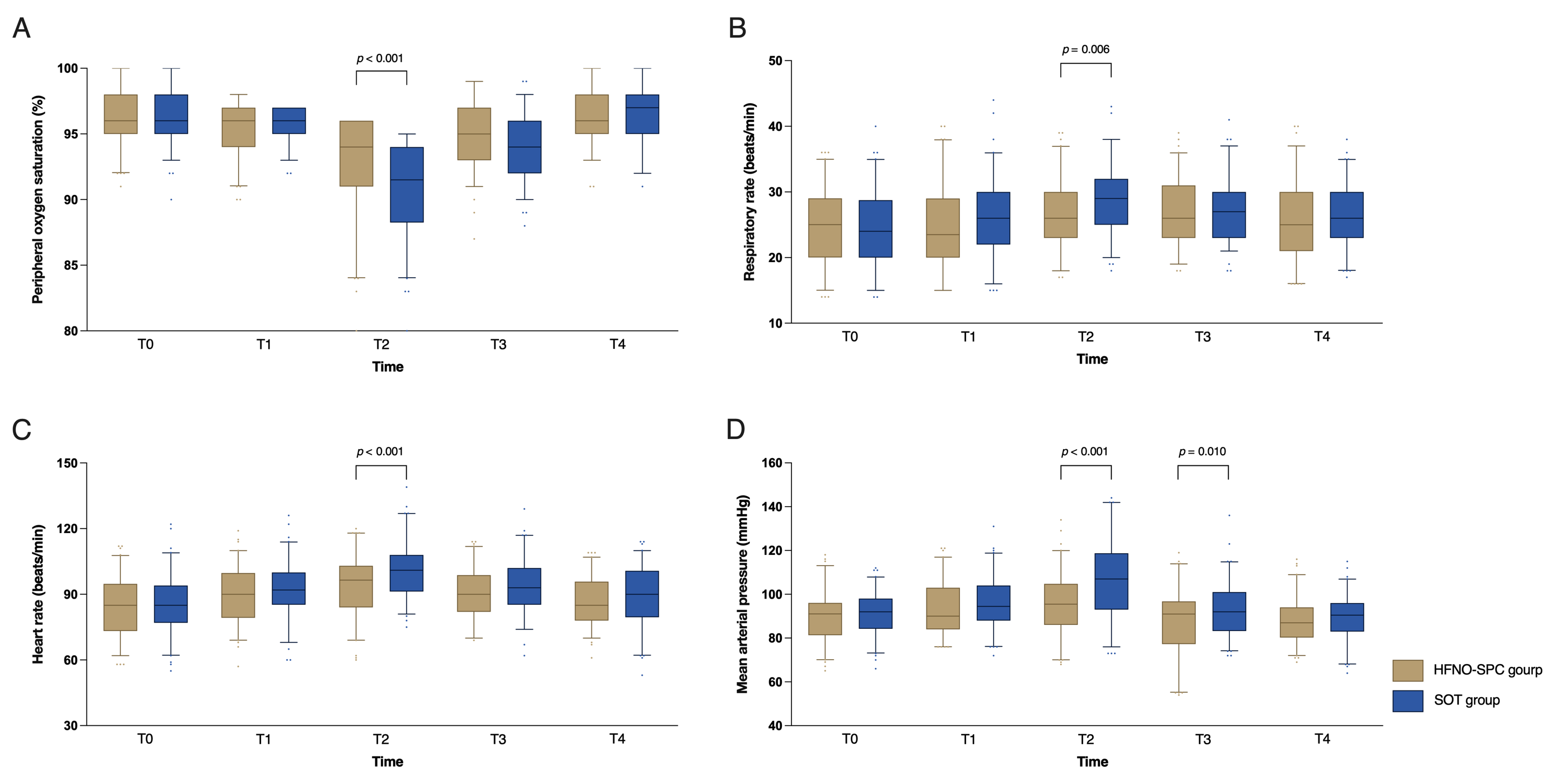


HFNO-SPC high-flow nasal oxygen therapy via a single-prong cannula interface, SOT standard oxygen therapy

3.4 Figure S4 EIT images from two representative patients. EIT images were obtained from patients receiving HFNO-SPC **(A)** or SOT **(B)** at five predefined time points (T0-T4). The top row in each panel shows TIV, with blue indicating tidal changes. The bottom row displays ΔEELI, with orange and light blue indicating regional loss and increase in end-expiratory lung volume, respectively. Compared with the patient in the SOT group, the patient receiving HFNO-SPC showed smaller decreases in both TIV and ΔEELI during and after FB.


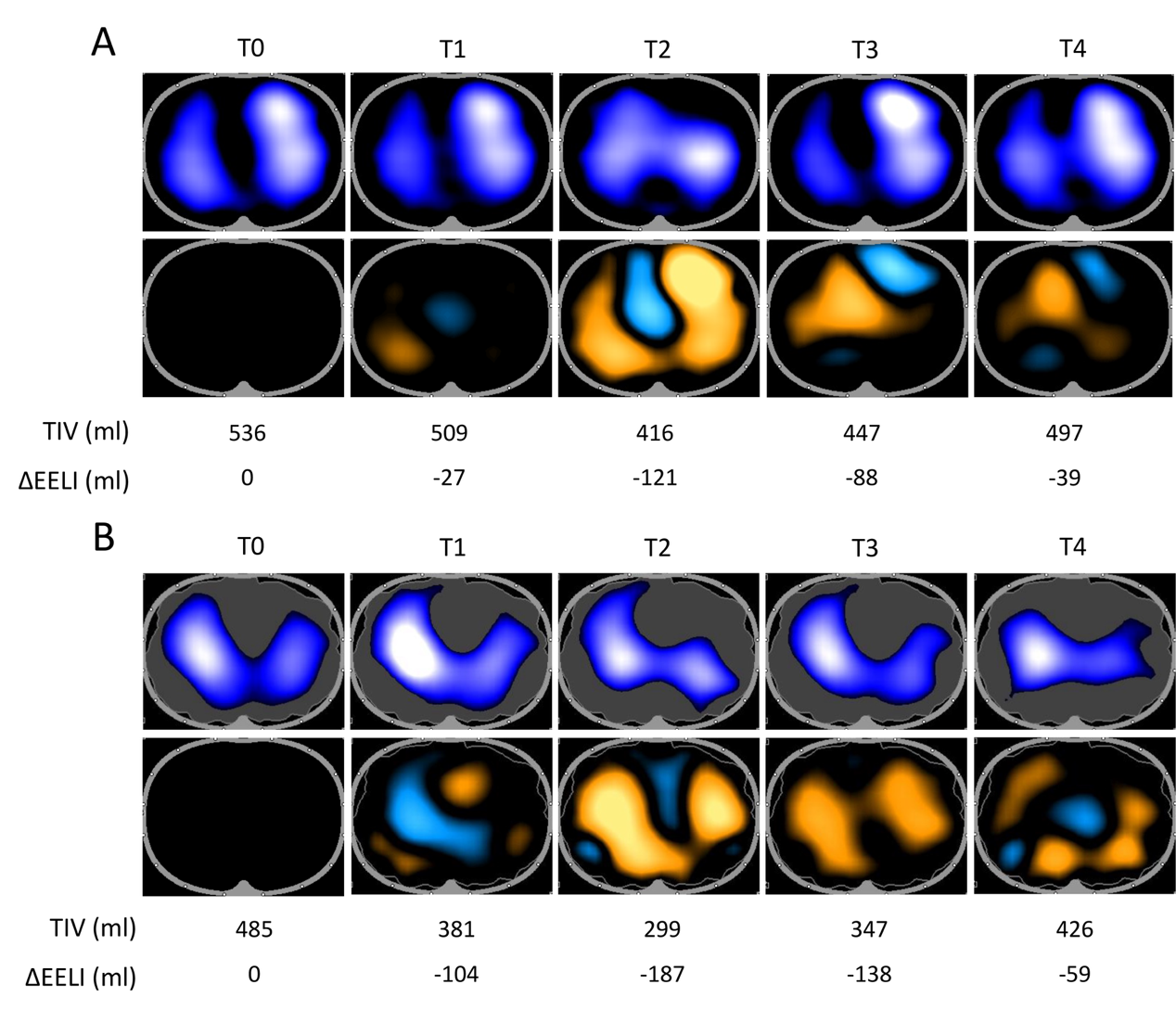


EIT electrical impedance tomography, HFNO-SPC high-flow nasal oxygen therapy via a single-prong cannula interface, SOT standard oxygen therapy, TIV, tidal impedance variation, ΔEELI the changes in end-expiratory lung impedance

3.5 Figure S5 Predicted probability of respiratory support escalation within 24 hours after FB based on ΔEELI measured at T3.


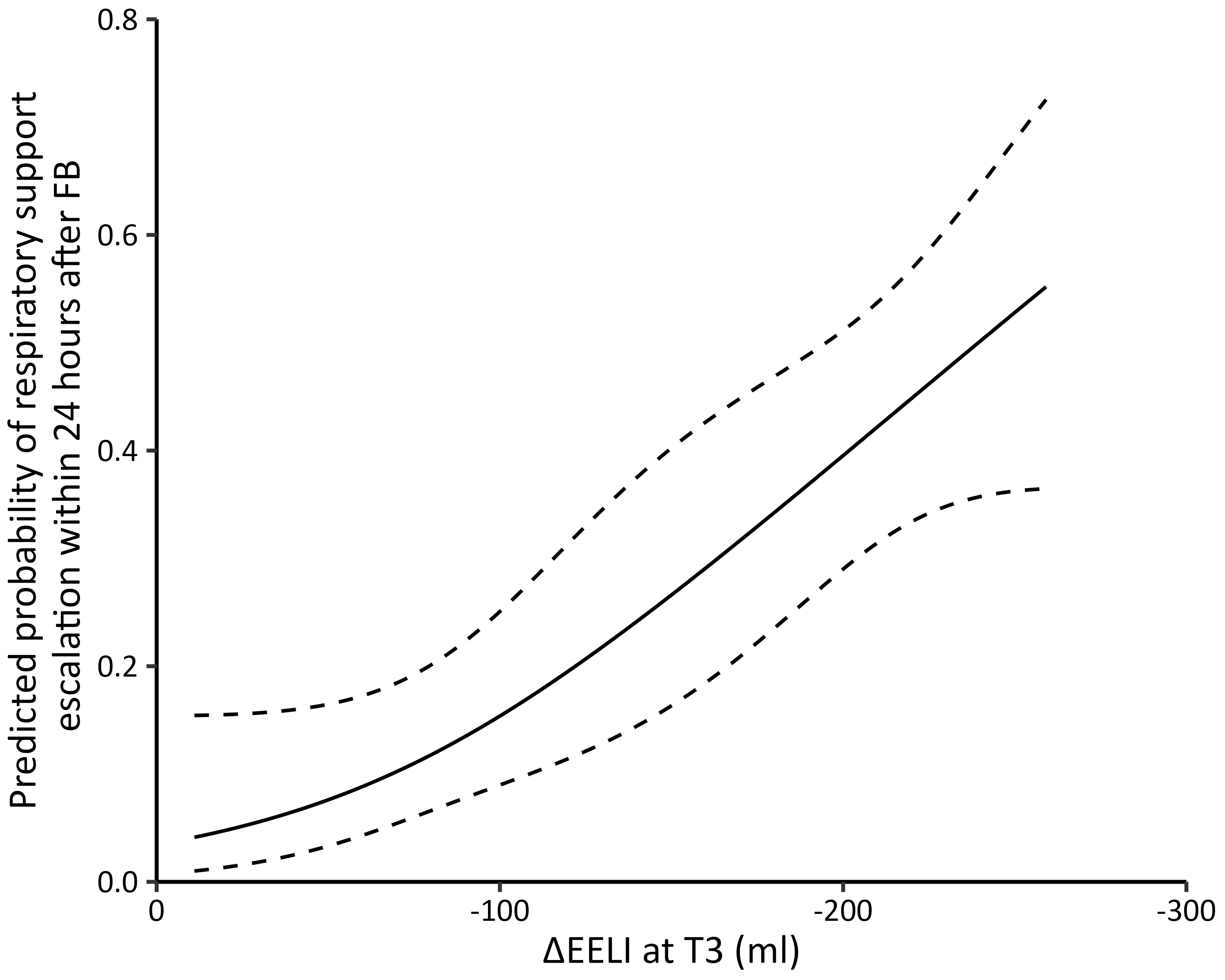


FB flexible bronchoscopy, ΔEELI the changes in end-expiratory lung impedance

3.6 Figure S6 Predicted probability of respiratory support escalation within 24 hours after FB based on ΔEELI measured at T4.


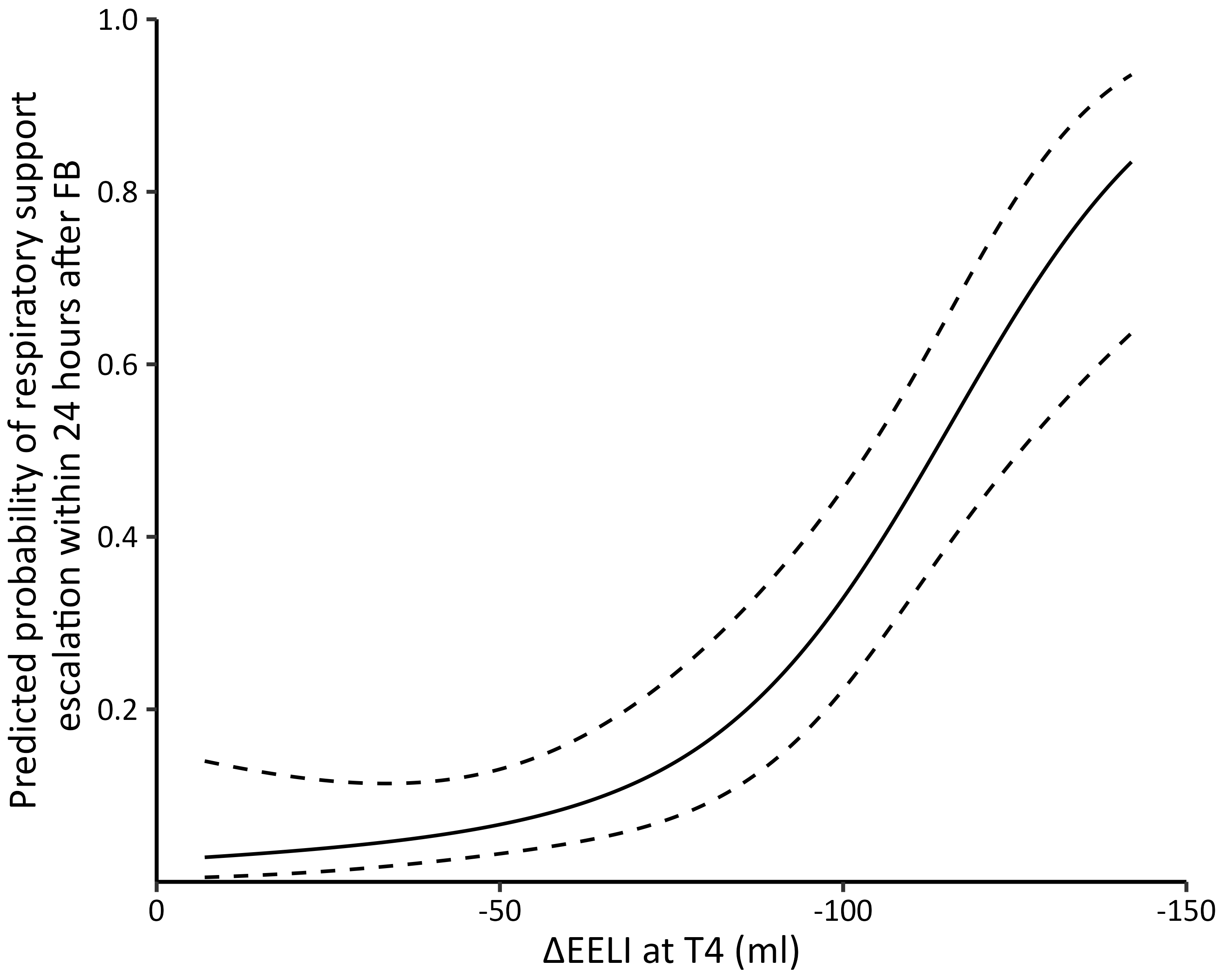


FB flexible bronchoscopy, ΔEELI the changes in end-expiratory lung impedance

3.7 Figure S7 Comparison of PaO_2_ **(A)**, PaCO_2_ **(B)**, and PaO_2_/FiO_2_ **(C)** between the HFNO-SPC group and the SOT group at T0 and T4.


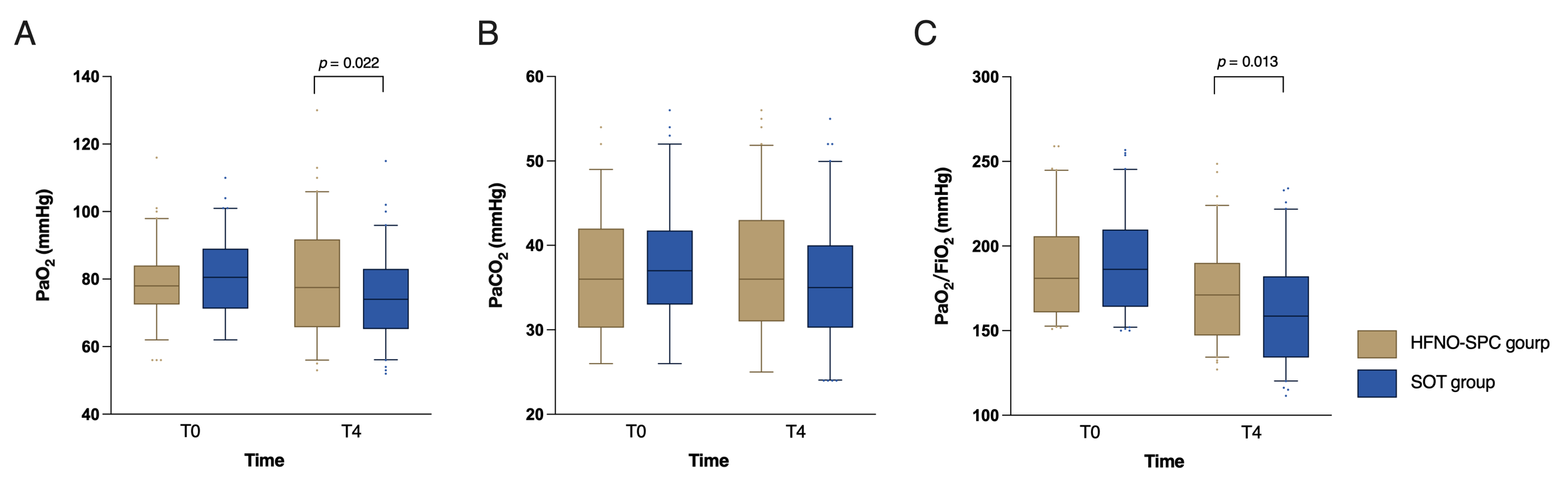


PaO_2_ partial pressure of arterial oxygen, PaCO_2_ partial pressure of arterial carbon dioxide, PaO_2_/FiO_2_ the ratio of the partial pressure of arterial oxygen to the fraction of inspired oxygen, HFNO-SPC high-flow nasal oxygen therapy via a single-prong cannula interface, SOT standard oxygen therapy

References

1. Wang R, Li HC, Li XY, Tang X, Chu HW, Yuan X, Tong ZH, Sun B: **Modified high-flow nasal cannula oxygen therapy versus conventional oxygen therapy in patients undergoing bronchoscopy: a randomized clinical trial**. *BMC Pulm Med* 2021, **21**(1):367.

2. Frat JP, Thille AW, Mercat A, Girault C, Ragot S, Perbet S, Prat G, Boulain T, Morawiec E, Cottereau A *et al*: **High-flow oxygen through nasal cannula in acute hypoxemic respiratory failure**. *N Engl J Med* 2015, **372**(23):2185-2196.
