# Supplementary material for: High-flow nasal oxygen therapy via a single-prong cannula interface during bronchoscopy in patients with acute respiratory failure: a two-center, open-label, randomized controlled trial
Source: Ann Intensive Care. 2026 May 12;16:100081. doi: 10.1016/j.aicoj.2026.100081 (PMC13195340; doi:10.1016/j.aicoj.2026.100081)
Supplement: Supplementary file 2 [file mmc2.docx]

**ADDITIONAL FILE 2: STUDY PROTOCOL**

**TRIAL SUMMARY**

Bronchoscopy is a valuable diagnostic and therapeutic tool but poses a significant risk of exacerbating respiratory failure in critically ill patients. Hypoxemia and increased respiratory support requirements are common complications. High-flow nasal cannula (HFNC) oxygen therapy provides stable oxygenation and reduced respiratory distress. Traditional HFNC interfaces, however, limit bronchoscopy due to the large-bore nasal cannula occupying both nostrils. We developed a modified HFNC oxygen therapy using a single-prong cannula interface to facilitate nasal bronchoscopy. This trial aims to demonstrate whether modified HFNC oxygen therapy reduces respiratory support escalation post-bronchoscopy in patients with acute respiratory failure (ARF) compared to standard oxygen therapy (SOT).

**OBJECTIVES**

**Primary Outcome**

To determine whether modified HFNC oxygen therapy, using a single-prong cannula interface, reduces the need for escalation of respiratory support within 24 hours after bronchoscopy in patients with ARF compared with standard oxygen therapy (SOT).

Escalation was defined as meeting any of the following criteria:

1. Requirement for invasive mechanical ventilation (IMV);

2. Requirement for non-invasive ventilation (NIV);

3. Requirement for HFNC oxygen therapy;

4. An increase in support parameters without changing the level of respiratory support: For NIV patients: a >20% increase in inspiratory positive airway pressure (IPAP), expiratory positive airway pressure (EPAP), or the fraction of inspired oxygen (FiO_2_); For HFNC oxygen therapy patients: a >20% increase in flow rate or FiO_2_; For low-flow nasal cannula oxygen therapy patients: a >50% increase in oxygen flow rate.

**Secondary Outcome**

Respiratory support escalation within 24 hours post-bronchoscopy will be further evaluated in a prespecified fixed-sequence hierarchical analysis.

**Other outcomes**

1. To evaluate whether modified HFNC oxygen therapy reduces the rate of endotracheal intubation at 24 hours, 7 days, and 28 days after bronchoscopy.

2. To assess whether modified HFNC oxygen therapy improves oxygenation stability, as measured by peripheral oxygen saturation (SpO_2_) levels during bronchoscopy.

3. To determine whether the use of modified HFNC oxygen therapy is associated with a reduced incidence of procedural interruptions during bronchoscopy.

4. To assess whether modified HFNC oxygen therapy shortens the procedure duration compared with SOT.

5. To assess the impact of modified HFNC oxygen therapy on ICU and hospital length of stay.

6. To evaluate whether modified HFNC oxygen therapy reduces all-cause mortality at 28 days, 60 days, and 90 days.

**PATIENT SELECTION CRITERIA**

Eligible patients must fulfill all inclusion criteria and none of the exclusion criteria.

**Inclusion Criteria:**

1. Adult patients aged 18 years or older;

2. Diagnosis of acute respiratory failure, defined by a ratio of the partial pressure of arterial oxygen to the fraction of inspired oxygen (PaO_2_/FiO_2_) less than 300 mmHg;

3. Clinical indication for bronchoscopy.

**Exclusion Criteria:**

1. Patients who are already intubated or have a tracheostomy in place at the time of screening.

2. Patients requiring immediate endotracheal intubation prior to bronchoscopy.

3. Patients with severe hypoxemia, defined as a PaO₂/FiO₂ ratio below 150 mmHg despite optimized supplemental oxygen therapy.

4. Patients with severe thrombocytopenia, defined as a platelet count less than 60 × 10^9^/L.

5. Patients with a recent acute myocardial infarction (within the preceding 6 weeks).

6. Patients with anatomical abnormalities or pathological conditions resulting in nasopharyngeal obstruction that preclude safe application of the nasal interface.

8. Patients with significant chest wall skin lesions or other contraindications that prevent the safe placement or accurate function of EIT monitoring.

9. Patients with known intolerance to HFNC oxygen therapy.

**STUDY DESIGN**

This study is a prospective, two-center, open-label, randomized controlled trial designed to evaluate the effectiveness and safety of modified HFNC oxygen therapy using a single-prong cannula interface compared with standard oxygen therapy (SOT) during bronchoscopy in patients with ARF.

The trial will be conducted in the ICUs of two tertiary hospitals and will enroll adult patients with acute respiratory failure who require diagnostic or therapeutic bronchoscopy. Eligibility will be assessed based on predefined inclusion and exclusion criteria, and written informed consent will be obtained from all participants or their legal representatives prior to enrollment.

Eligible patients will be randomly assigned in a 1:1 ratio to receive either modified HFNC oxygen therapy or SOT during the bronchoscopy procedure. Randomization will be stratified by study site to ensure balanced allocation across centers.

Due to the visible differences between the oxygen delivery devices, blinding of participants and treating clinicians is not feasible. To minimize potential assessment bias, outcome evaluation and statistical analysis will be performed by independent statistician blinded to treatment allocation.

**INTERVENTIONS**

**1. Modified HFNC oxygen therapy group**

Participants in this group will receive oxygen therapy via the AIRVO 2 system (Fisher & Paykel Healthcare, New Zealand) using a single-prong nasal cannula interface. The size of the nasal cannula was selected according to the patient’s nostrils to ensure proper fit and comfort. The system will deliver heated and humidified oxygen at an initial flow rate of 60 L/min and a fraction of inspired oxygen (FiO_2_) set at 0.8, titrated as needed up to 1.0 to maintain SpO_2_ above 90% during the procedure. All participants will remain on the assigned oxygen modality during the entire bronchoscopy procedure and for post-procedural observation until clinical stabilization.

**2. SOT group**

Participants in the control group will receive oxygen via a standard non-rebreathing reservoir mask with a flow rate of 10-15 L/min, titrated to maintain SpO_2_ above 90% during the procedure. The mask will remain in place throughout the bronchoscopy procedure. Oxygen therapy will continue until the patient is clinically stable following bronchoscopy.

**STUDY PROCEDURES**

The study will be conducted in a stepwise and standardized manner to ensure consistency across participating centers and to maintain patient safety. The key steps of the study procedures are outlined below:

**Step 1: Patient screening and enrollment**

Patients admitted to the ICU with AFR requiring bronchoscopy will be screened based on predefined inclusion and exclusion criteria. Eligible patients or their legal representatives will be approached for informed consent, with adequate explanation of study objectives, procedures, risks, and benefits.

**Step 2: Baseline assessment**

Collection of demographic data, medical history, and baseline clinical parameters, including vital signs, arterial blood gas analysis, and routine laboratory results.

**Step 3: Randomization**

Patients will be randomly allocated in a 1:1 ratio to either the modified HFNC group or the SOT group. Randomization will be stratified by study site using a computer-generated sequence and allocation will be concealed with sealed opaque envelopes.

**Step 4: Pre-bronchoscopy preparation**

The assigned oxygen delivery modality (modified HFNC or SOT) will be applied, titrated to maintain SpO_2_ >90%.

Continuous monitoring of vital signs and arterial blood gas analysis will be performed. A properly sized 16-electrode EIT belt will be placed around the patient’s chest, just below the axillae at the level of the fourth to fifth intercostal spaces. The belt position will be marked to prevent displacement during the study. The EIT belt will remain in place for continuous monitoring throughout the procedure.

Sedation will be provided using intravenous midazolam (0.05-0.1 mg/kg) and/or propofol (up to 1 mg/kg) based on patient needs. Topical anesthesia will be administered to the nasal and oropharyngeal mucosa.

**Step 5: Bronchoscopy procedure**

Bronchoscopy will be performed via nasal access following standard clinical practice. Diagnostic interventions such as bronchoalveolar lavage (BAL), bronchial brushing, endobronchial biopsy, or transbronchial lung biopsy will be performed when clinically indicated. Any procedural interruption or adverse events will be documented in real time. Vital signs and EIT parameters will be recorded at five predefined time points: T0, before bronchoscopy; T1, upon insertion of the bronchoscope into the nasal cavity; T2, at the end of the procedure; T3, 10 minutes after bronchoscopy; and T4, 2 hours after bronchoscopy.

**Step 6: Immediate post-procedure care**

Participants will continue on their allocated oxygen therapy during the recovery period. EIT measurements and arterial blood gas analysis will be performed at 2 hours after bronchoscopy. Patients will be closely monitored for respiratory deterioration or need for escalation of respiratory support.

**Step 7: Follow-up and outcome assessment**

Patients will be followed for up to 90 days to evaluate all primary and secondary outcomes, including short-term respiratory events and longer-term mortality.

**DATA COLLECTION**

Data will be collected prospectively using standardized case report forms (CRFs) specifically designed for this study. Trained research staff at each participating center will be responsible for accurate and timely data entry.

**1. Demographics and baseline characteristics**

(1) Age, sex, body mass index (BMI);

(2) Severity scores: APACHE II, SOFA;

(3) Smoking history;

(4) Comorbidities;

(5) Indication for bronchoscopy;

(6) Respiratory support prior to bronchoscopy;

(7) Baseline vital signs;

(8) Arterial blood gas analysis.

**2. Procedural data**

(1) Type of bronchoscopy and diagnostic interventions: BAL, bronchial brushing, endobronchial biopsy, transbronchial lung biopsy;

(2) Volume of BAL fluid instilled and recovered;

(3) Duration of bronchoscopy;

(4) Vital signs, including respiratory rate, heart rate, mean arterial pressure, and SpO_2_, as well as EIT measurements such as tidal impedance variation (TIV) and changes in end-expiratory lung impedance (ΔEELI), will be recorded at five predefined time points: T0, T1, T2, T3, and T4;

(5) Arterial blood gas analysis at T4.

**3. Intra-bronchoscopy and post-bronchoscopy events**

(1) Events during bronchoscopy: agitation, bronchospasm, arrhythmias/tachycardia ≥150 bpm, hypertension, epistaxis, mucosal bleeding;

(2) Events within 24 hours post-bronchoscopy: transient fever, pneumothorax, hemorrhage;

(3) Lowest SpO_2_ recorded during bronchoscopy;

(4) Number of procedure interruptions.

**4. Clinical Outcomes**

(1) Respiratory support escalation within 24 hours after bronchoscopy;

(2) Intubation rates within 24 hours, 7 days, and 28 days;

(3) ICU length of stay;

(4) Hospital length of stay;

(5) All-cause mortality at 28, 60, and 90 days.

**STATISTICAL ANALYSIS**

All statistical analyses will be performed according to the predefined Statistical Analysis Plan (SAP). Analyses will follow the intention-to-treat principle, with additional per-protocol analyses conducted as appropriate.

Descriptive statistics will be used to summarize baseline characteristics. Categorical variables will be compared using the chi-square test or Fisher’s exact test, and continuous variables will be analyzed using t-tests or Mann–Whitney U tests, as appropriate.

The primary outcome (escalation of respiratory support within 24 hours) will be analyzed using logistic regression The key secondary outcome (respiratory support escalation assessed hierarchically) will be analyzed using the win ratio method in a prespecified fixed-sequence hierarchical framework to prioritize endpoints and control for multiplicity. Time-to-event outcomes will be analyzed using Kaplan–Meier survival curves and log-rank tests.

All statistical tests will be two-sided, and a p-value <0.05 will be considered statistically significant.

Details of sample size calculation, sensitivity analyses, and handling of missing data are provided in the SAP.

**DATA MANAGEMENT**

All study data will be recorded using standardized CRFs by trained investigators at each site. Data will be securely stored, anonymized, and subjected to regular quality checks and audits to ensure accuracy and completeness. Any protocol deviations will be clearly documented, reviewed, and addressed in accordance with Good Clinical Practice (GCP) guidelines.

**INFORMED CONSENT PROCESS**

Written informed consent will be obtained from all participants or their legally authorized representatives prior to enrollment. The consent process will include a clear explanation of study procedures, potential risks, benefits, and the right to withdraw at any time without affecting standard care. Emergency consent procedures will be available if participants are temporarily unable to provide consent.

**ETHICAL CONSIDERATIONS**

All study procedures will adhere to the principles of the Declaration of Helsinki and relevant ethical guidelines. Risk management procedures will be clearly defined and continuously monitored to minimize harm and ensure participant safety. The study will emphasize respect for patient autonomy, privacy, and the right to withdraw without prejudice.
