# Supplementary material for: High-flow nasal oxygen therapy via a single-prong cannula interface during bronchoscopy in patients with acute respiratory failure: a two-center, open-label, randomized controlled trial
Source: Ann Intensive Care. 2026 May 12;16:100081. doi: 10.1016/j.aicoj.2026.100081 (PMC13195340; doi:10.1016/j.aicoj.2026.100081)
Supplement: Supplementary file 3 [file mmc3.docx]

ADDITIONAL FILE 3: STATISTICAL ANALYSIS PLAN

Modified High-Flow Nasal Cannula Oxygen Therapy via a Single-Prong Cannula Interface During Bronchoscopy in Patients

with Acute Respiratory Failure:

A Two-Center, Open-Label, Randomized Controlled Trial

Contents

[1. Introduction 3](#_Toc204512856)

[2. Study design 5](#_Toc204512857)

[3. Study objective 6](#_Toc204512858)

[4. Endpoints 7](#_Toc204512859)

[4.1 Primary endpoint 7](#_Toc204512860)

[4.2 Secondary endpoint 7](#_Toc204512861)

[4.3 Vital Signs, Electrical Impedance Tomography, Arterial Blood Gas Analysis 8](#_Toc204512862)

[5. Study Population 10](#_Toc204512863)

[5.1 Inclusion criteria 10](#_Toc204512864)

[5.2 Exclusion criteria 10](#_Toc204512865)

[6. Sample Size Calculation 11](#_Toc204512866)

[7. Statistical analysis 12](#_Toc204512867)

[7.1 Basic principles 12](#_Toc204512868)

[7.2 Primary Endpoint Analysis 12](#_Toc204512869)

[7.3 Secondary Endpoint Analysis 13](#_Toc204512870)

[7.4 Vital Signs, Electrical Impedance Tomography, and Arterial Blood Gas Analysis 14](#_Toc204512871)

[7.5 Subgroup analysis 16](#_Toc204512872)

[7.6 Missing data 16](#_Toc204512873)

[8. Interim Analyses and Data Monitoring 17](#_Toc204512874)

[9. Multiplicity considerations 18](#_Toc204512875)

[10. Tables 19](#_Toc204512876)

[11. References 30](#_Toc204512877)

# 1. Introduction

Bronchoscopy plays a critical role in the diagnosis and management of respiratory failure. However, maintaining adequate oxygenation during the procedure remains challenging. Studies have reported that the partial pressure of arterial oxygen (PaO_2_) typically decreases by approximately 20 mmHg during bronchoscopy [1], with a greater decline seen in procedures involving bronchoalveolar lavage (BAL) [2]. In patients with acute respiratory failure, persistent hypoxemia may necessitate increasing the fraction of inspired oxygen (FiO_2_) or temporarily withdrawing the bronchoscope to allow for oxygenation recovery. Such interruptions can hinder the completion of essential diagnostic procedures, including bronchoalveolar lavage, brushing, mucosal biopsy, or transbronchial lung biopsy, and in severe cases, may result in emergent intubation and initiation of invasive mechanical ventilation (IMV).

Current respiratory support strategies during bronchoscopy include facemask oxygen therapy, noninvasive ventilation (NIV), and high-flow nasal cannula (HFNC) oxygen therapy. Facemask oxygen delivery fails to provide high or consistently controlled FiO_2_, while NIV often poses challenges related to patient tolerance and requires considerable operator expertise. HFNC provides stable FiO_2_ and low-level positive end-expiratory pressure (PEEP), but its traditional dual-nasal-prong interface can interfere with bronchoscope insertion and manipulation, limiting its utility during procedures.

To address these limitations, we developed a modified HFNC oxygen therapy using a single-prong cannula interface, which ensures effective oxygenation while allowing unobstructed access for bronchoscopy. This study aims to evaluate the clinical feasibility and effectiveness of this approach in patients with acute respiratory failure, focusing on the prevention of procedure-related deterioration and the reduction of respiratory support escalation.

# 2. Study design

Our study was a two-center, parallel-arm, open-label, prospective randomized controlled trial. Eligible patients were randomly assigned to receive either modified HFNC oxygen therapy using a single-prong cannula or standard oxygen therapy (SOT) delivered via a non-rebreathing reservoir mask. Prespecified clinical and physiological endpoints were assessed in both groups.

# 3. Study objective

To confirm that our developed modified HFNC oxygen therapy significantly reduces the need for respiratory support escalation after bronchoscopy compared with SOT in patients with acute respiratory failure.

# 4. Endpoints

## 4.1 Primary endpoint

The primary endpoint was the need for respiratory support escalation within 24 hours after bronchoscopy. Escalation was defined as meeting any of the following criteria:

- Requirement for IMV;
- Requirement for NIV;
- Requirement for HFNC oxygen therapy;
- An increase in support parameters without changing the level of respiratory support: For NIV patients: a >20% increase in inspiratory positive airway pressure (IPAP), expiratory positive airway pressure (EPAP), or FiO_2_; For HFNC oxygen therapy patients: a >20% increase in flow rate or FiO_2_; For low-flow nasal cannula oxygen therapy patients: a >50% increase in oxygen flow rate.

## 4.2 Secondary endpoint

The secondary endpoint was a hierarchical composite outcome, assessed in the following fixed sequence Since the primary endpoint is a composite endpoint, the secondary endpoints were defined as a hierarchy of progressively respiratory support forms based on clinical severity with the following prespecified order: (time frame: within 24 hours after bronchoscopy):

1. Requirement for IMV;
2. Requirement for NIV;
3. Requirement for HFNC oxygen therapy;
4. An increase in support parameters without changing the level of respiratory support.

Additional endpoints included:

- The need for endotracheal intubation within 24 hours after bronchoscopy;
- The need for endotracheal intubation within 7 days after bronchoscopy;
- The lowest oxygen saturation (SpO_2_) recorded during bronchoscopy;
- The number of patients requiring interruption of bronchoscopy;
- The duration of bronchoscopy;
- Intensive Care Unit (ICU) length of stay (time frame: from randomization to discharge from ICU);
- Hospital length of stay (time frame: from randomization to discharge from hospital);
- Mortality at 28, 60, and 90 days after bronchoscopy.

## 4.3 Vital Signs, Electrical Impedance Tomography, Arterial Blood Gas Analysis

- SpO_2_;
- Respiratory rate;
- Heart rate;
- Mean arterial pressure;
- Tidal impedance variation (TIV);
- Changes in end-expiratory lung impedance (ΔEELI).
- pH;
- PaO_2_;
- PaCO_2_;
- HCO_3_^-^;
- PaO_2_/FiO_2_.

# 5. Study Population

## 5.1 Inclusion criteria

- Age ≥ 18 years;
- Respiratory failure defined as PaO_2_/FiO_2_ <300 mm Hg;
- Clinical indication for bronchoscopy to diagnose or treat pulmonary disease.

## 5.2 Exclusion criteria

- Pre-existing endotracheal intubation or tracheostomy;
- Required immediate endotracheal intubation;
- PaO_2_/FiO_2_ <150 mm Hg;
- Platelet count <60 × 10^9^/L;
- History of myocardial infarction within the past 6 weeks;
- Nasopharyngeal obstruction or blockage;
- Presence of chest skin lesions contraindicating the application of electrical impedance tomography;
- Intolerance to modified HFNC oxygen therapy.

# 6. Sample Size Calculation

The sample size was calculated based on the primary hypothesis that modified HFNC oxygen therapy would reduce the incidence of respiratory support escalation within 24 hours after bronchoscopy compared with SOT in patients with respiratory failure. Based on previous clinical experience and published studies, it was estimated that approximately 35% of patients in the SOT group would require escalation of respiratory support [3]. Assuming this rate could be reduced to 15% in the modified HFNC oxygen therapy, a total of 144 patients would be required to detect this difference with 80% power at a two-sided alpha level of 0.05. To account for 10% of potential dropouts, the final enrollment target was set at 160 patients.

# 7. Statistical analysis

## 7.1 Basic principles

Both primary and secondary endpoint analyses will adhere to the intention-to-treat (ITT) principle. No post-randomization exclusions will be applied except for participants who withdraw consent. Concurrently, a per-protocol (PP) analysis will be conducted and reported, including all participants who completed key interventions and maintained adequate treatment compliance.

Categorical variables are presented as frequencies (percentages). Continuous variables are summarized as mean ± standard deviation when approximately normally distributed; otherwise as median (interquartile range, IQR).

## 7.2 Primary Endpoint Analysis

For the primary endpoint comparison between groups, the χ² test will be used when validity conditions are met; otherwise, Fisher's exact test will be employed. The analysis will report risk differences with 95% confidence intervals (CI), calculated using the Wald method when both group proportions fall between 0.2-0.8 with frequencies >10 per cell, otherwise using the Miettinen-Nurminen method [4, 5].

Subsequently, a Cox proportional hazards model will be utilized to evaluate the effect of treatment allocation (HFNC vs. SOT) on the primary outcome. Results will be reported as hazard ratios (HR) with corresponding 95% confidence intervals (CI).Cumulative incidence for the primary endpoint with time-to-event data will be estimated for the two groups using the Kaplan-Meier method and compared using a log-rank test.

## 7.3 Secondary Endpoint Analysis

The secondary endpoint of respiratory support escalation within 24 hours after bronchoscopy was analyzed using the unmatched win statistics method. This composite outcome consisted of four hierarchically ordered components based on clinical relevance: (1) requirement for IMV, (2) requirement for NIV, (3) requirement for HFNC therapy, and (4) an increase in support parameters without a change in the level of respiratory support.

Consider enrolling N_t_ and N_c_ subjects in the HFNC and SOT arms respectively, generating N_t_ × N_c_ possible pairs. Let: i = 1, ..., N_t_ denote the i-th subject in the HFNC group; l = 1, ..., N_c_ denote the l-th subject in the SOT group. The kernel functions K and L are defined as: K_il_ = 1 if the HFNC subject i wins against the SOT subject l, otherwise 0;L_il_ = 1 if the SOT subject l wins against the HFNC subject i, otherwise 0. The algorithm is as follows:

Step 1: Compare all pairs for secondary outcome (1):

HFNC wins if min(T_trt_, C_trt_, C_con_) > T_con_;

SOT wins if min(T_con_, C_con_, C_trt_) > T_trt_;

Otherwise, the pair is tied.

(T_trt_: Event time in the HFNC group; C_trt_: Censoring time in the HFNC group; T_con_: Event time in the SOT group; C_con_: Censoring time in the SOT group)

Step 2: For tied pairs, compare the next hierarchical outcome;

Step 3: Repeat Step 2 until all outcomes are evaluated.

Based on the counting approach, we define the win proportions Pt and Pc:

$$P_{t}=\frac{\sum_{i=1}^{N_{t}} \sum_{l=1}^{N_{c}} K_{il}}{N_{t}N_{c}}\text{ and }P_{c}=\frac{\sum_{i=1}^{N_{t}} \sum_{l=1}^{N_{c}} L_{il}}{N_{t}N_{c}}$$

The win statistics are defined based on P_t_ and P_c_ as:

Win Ratio: WR = P_t_/P_c_

Net benefit: NB = P_t_-P_c_

The variance estimates and associated p-values were calculated according to the methodology established by Dong et al [6].

For other endpoints, categorical variables will be analyzed identically to the primary endpoint. Continuous variables will be compared using Student's t-test (normally distributed data) or Mann-Whitney U test (non-normally distributed data), with absolute differences reported(median differences corresponding 95% confidence intervals derived from 5,000 bootstrap resamples). Treatment effects will be assessed using logistic regression for binary outcomes or Cox proportional hazards models for time-to-event outcomes.

7.4 Vital Signs, Electrical Impedance Tomography, and Arterial Blood Gas Analysis

A two-factor (treatment group and time point) repeated-measures ANOVA will be used to compare differences between the two groups in the following parameters at five predefined time points: T0 (before bronchoscopy), T1 (upon insertion of the bronchoscope into the nasal cavity), T2 (at the end of bronchoscopy), T3 (10 minutes after bronchoscopy), and T4 (2 hours after bronchoscopy):

- SpO_2_;
- Respiratory rate;
- Heart rate;
- Mean arterial pressure;
- TIV;
- ΔEELI.

Where necessary, the Bonferroni adjustment for multiple testing will be applied to post-hoc comparisons across repeated time points to ensure that the family-wise error rate does not exceed 0.05. A restricted cubic spline logistic regression model was used to assess the association between ΔEELI at T2, T3, and T4 and the escalation of respiratory support within 24 hours following bronchoscopy. Knots were placed at the 10th, 50th, and 90th percentiles of each variable. Additionally, at T0 and T4 time points, compare the following arterial blood gas parameters between the HFNC group and the SOT group:

- pH;
- PaO_2_;
- PaCO_2_;
- HCO_3_^-^;
- PaO_2_/FiO_2_.

## 7.5 Subgroup analysis

We will conduct subgroup analyses for the primary endpoint based on the following stratification factors:

- Study center (Chao-Yang, Qinghai);
- Age group (<60 years, ≥60 years);
- Sex (Male, Female);
- APACHE II score at randomization (<Median, ≥Median)
- Immunocompromised status at randomization (Yes, No)
- PaO_2_:FiO_2_ ratio at randomization (<200 mm Hg, ≥200 mm Hg)
- ΔEELI at T2 (end of procedure) (<Median, ≥Median)
- Amount of fluid instilled (<Median mL, ≥Median mL).

Subgroup analyses will employ the same analytical methods as used for the primary endpoint measure. To evaluate the interaction effect (including 95% confidence interval and p-value), the subgroup variable and its interaction term with the treatment group will be incorporated into the main regression model.

## 7.6 Missing data

We anticipate no missing data for the primary outcome measure. For secondary or exploratory outcomes with missing data points, a complete case analysis will be employed, including only cases with complete data for the outcome variable.

# 8. Interim Analyses and Data Monitoring

Given the relatively short follow-up duration of this trial and the established safety profile of the intervention, no interim analyses are planned. The developed modified HFNC oxygen therapy is not anticipated to pose greater harm than standard oxygen therapy, and therefore, no formal statistical stopping rules or predefined early termination criteria have been specified.

# 9. Multiplicity considerations

We did not correct for multiple comparisons for secondary outcomes or subgroup analyses; accordingly, the findings from these analyses should be interpreted as exploratory.

# 10. Tables

Table 1. Characteristics of patients at randomization

| **Characteristics** | **All patients** | **Modified HFNC oxygen group** | **SOT group** |
| --- | --- | --- | --- |
| **Age, years** |  |  |  |
| **Male, no. (%)** |  |  |  |
| **Body mass index, kg/m^2^** |  |  |  |
| **APACHE II score** |  |  |  |
| **SOFA score** |  |  |  |
| **Smoking history , no. (%)** |  |  |  |
| Ever smoked |  |  |  |
| Currently smoke |  |  |  |
| **Comorbidities, no. (%)** |  |  |  |
| Immunocompromised |  |  |  |
| COPD or asthma |  |  |  |
| Coronary artery disease |  |  |  |
| Hypertension |  |  |  |
| Diabetes mellitus |  |  |  |
| Chronic renal insufficiency |  |  |  |
| Cerebrovascular disease |  |  |  |
| **Indication for** **bronchoscopy, no. (%)** |  |  |  |
| Severe community-acquired pneumonia |  |  |  |
| Suspected hospital-acquired pneumonia |  |  |  |
| Pneumonia in immunocompromised host |  |  |  |
| Interstitial lung disease |  |  |  |
| Suspected lung cancer |  |  |  |
| Hemoptysis |  |  |  |
| **Respiratory support before bronchoscopy, no. (%)** |  |  |  |
| LFNC oxygen therapy |  |  |  |
| HFNC |  |  |  |
| NIV |  |  |  |
| **Vital signs** |  |  |  |
| Temperature, °C |  |  |  |
| Respiratory rate, beats/min |  |  |  |
| Heart rate, beats/min |  |  |  |
| SpO_2_, % |  |  |  |
| Mean arterial pressure, mmHg |  |  |  |
| **Arterial blood gas** |  |  |  |
| pH |  |  |  |
| PaO_2_, mmHg |  |  |  |
| PaCO_2_, mmHg |  |  |  |
| HCO_3_^-^, mmol/L |  |  |  |
| PaO_2_/FiO_2_, mmHg |  |  |  |
| **Laboratory tests** |  |  |  |
| White blood cell, ×10^9^/L |  |  |  |
| Neutrophil, ×10^9^/L |  |  |  |
| Lymphocyte, ×10^9^/L |  |  |  |
| Hemoglobin, g/L |  |  |  |
| Hematocrit, % |  |  |  |
| Platelet, ×10^9^/L |  |  |  |
| Albumin, g/L |  |  |  |
| Aspartate aminotransferase, U/L |  |  |  |
| Alanine aminotransferase, U/L |  |  |  |
| Total bilirubin, μmol/L |  |  |  |
| Direct bilirubin, μmol/L |  |  |  |
| Blood urea nitrogen, mmol/L |  |  |  |
| Creatinine, μmol/L |  |  |  |
| Sodium, mmol/L |  |  |  |
| Potassium, mmol/L |  |  |  |
| Glucose, mmol/L |  |  |  |
| Prothrombin time, s |  |  |  |
| Activated partial thromboplastin time, s |  |  |  |
| Fibrinogen, mg/dl |  |  |  |
| D-Dimer, mg/L |  |  |  |
| N-terminal pro B-type natriuretic peptide, pg/ml |  |  |  |
| C-reaction protein, mg/L |  |  |  |
| Procalcitonin, ng/ml |  |  |  |

HFNC high-flow nasal cannula, SOT standard oxygen therapy, APACHE II Acute Physiology and Chronic Health Evaluation II, SOFA sequential organ failure assessment, COPD chronic obstructive pulmonary disease, LFNC low-flow nasal cannula, NIV noninvasive ventilation, SpO_2_ peripheral capillary oxygen saturation, PaO_2_ partial pressure of arterial oxygen, PaCO_2_ partial pressure of arterial carbon dioxide, HCO_3_^-^ bicarbonate, FiO_2_ fraction of inspired oxygen

Table 2. Clinical endpoints according to the intention-to-treat principle

| **Endpoints** | **Modified HFNC oxygen group** | **SOT group** | **Mean, median, or risk difference, (95% CI)** | **Relative difference, (95% CI)** | ***P*** |
| --- | --- | --- | --- | --- | --- |
| **Primary endpoint** |  |  |  |  |  |
| Respiratory support escalation within 24h after bronchoscopy, no. (%) |  |  |  |  |  |
| **Secondary endpoint** |  |  |  |  |  |
| Respiratory support escalation within 24h after bronchoscopy tested in prespecified fixed sequence, no. (%) |  |  |  |  |  |
| **Other endpoints** |  |  |  |  |  |
| Intubation within 24h after bronchoscopy, no. (%) |  |  |  |  |  |
| Intubation within 7d after bronchoscopy, no. (%) |  |  |  |  |  |
| Intubation within 28d after bronchoscopy, no. (%) |  |  |  |  |  |
| Lowest SpO_2_ during bronchoscopy, % |  |  |  |  |  |
| Number of patients with interrupted bronchoscopy, no. (%) |  |  |  |  |  |
| Duration of bronchoscopy, min |  |  |  |  |  |
| ICU length of stay, days |  |  |  |  |  |
| Hospital length of stay, days |  |  |  |  |  |
| 28-day mortality, no. (%) |  |  |  |  |  |
| 60-day mortality, no. (%) |  |  |  |  |  |
| 90-day mortality, no. (%) |  |  |  |  |  |

HFNC high-flow nasal cannula, SOT standard oxygen therapy, SpO_2_ peripheral capillary oxygen saturation, ICU intensive care unit

Table 3. Clinical endpoints according to the per-protocol principle

| **Endpoints** | **Modified HFNC oxygen group** | **SOT group** | **Mean, median, or risk difference, (95% CI)** | **Relative difference, (95% CI)** | ***P*** |
| --- | --- | --- | --- | --- | --- |
| **Primary endpoint** |  |  |  |  |  |
| Respiratory support escalation within 24h after bronchoscopy, no. (%) |  |  |  |  |  |
| **Secondary endpoint** |  |  |  |  |  |
| Respiratory support escalation within 24h after bronchoscopy tested in prespecified fixed sequence, no. (%) |  |  |  |  |  |
| **Other endpoints** |  |  |  |  |  |
| Intubation within 24h after bronchoscopy, no. (%) |  |  |  |  |  |
| Intubation within 7d after bronchoscopy, no. (%) |  |  |  |  |  |
| Intubation within 28d after bronchoscopy, no. (%) |  |  |  |  |  |
| Lowest SpO_2_ during bronchoscopy, % |  |  |  |  |  |
| Number of patients with interrupted bronchoscopy, no. (%) |  |  |  |  |  |
| Duration of bronchoscopy, min |  |  |  |  |  |
| ICU length of stay, days |  |  |  |  |  |
| Hospital length of stay, days |  |  |  |  |  |
| 28-day mortality, no. (%) |  |  |  |  |  |
| 60-day mortality, no. (%) |  |  |  |  |  |
| 90-day mortality, no. (%) |  |  |  |  |  |

HFNC high-flow nasal cannula, SOT standard oxygen therapy, SpO_2_ peripheral capillary oxygen saturation, ICU intensive care unit

Table 4. Respiratory support escalation within 24h after bronchoscopy in the modified HFNC oxygen group and SOT groups

|  | **All patients** | **Modified HFNC oxygen group** | **SOT group** | ***P*** |
| --- | --- | --- | --- | --- |
| Maintained the same respiratory support level |  |  |  |  |
| LFNC oxygen therapy, then HFNC oxygen therapy |  |  |  |  |
| LFNC oxygen therapy, then HFNC oxygen therapy, then NIV |  |  |  |  |
| HFNC oxygen therapy, then flow rate or FiO_2_ increase > 20% |  |  |  |  |
| HFNC oxygen therapy, then NIV |  |  |  |  |
| HFNC oxygen therapy, then NIV, then IMV |  |  |  |  |
| NIV, then IPAP, EPAP, or FiO_2_ increase > 20% |  |  |  |  |
| NIV, then IMV |  |  |  |  |

HFNC high-flow nasal cannula, SOT standard oxygen therapy, LFNC low-flow nasal cannula, NIV noninvasive ventilation, FiO_2_ fraction of inspired oxygen, IMV invasive mechanical ventilation, IPAP inspiratory positive airway pressure, EPAP expiratory positive airway pressure

Table 5. Comparison of vital signs and EIT measurements between the modified HFNC oxygen group and SOT groups

| **Variables** | **Group** | **T0** | **T1** | **T2** | **T3** | **T4** | ***P*** |
| --- | --- | --- | --- | --- | --- | --- | --- |
| **Vital signs** |  |  |  |  |  |  |  |
| SpO_2_, % | Modified HFNC |  |  |  |  |  | *p^a^* |
|  | SOT |  |  |  |  |  | *p^a^* |
|  | *P^c^* |  |  |  |  |  | *p^b^* |
| Respiratory rate, beats/min | Modified HFNC |  |  |  |  |  | *p^a^* |
|  | SOT |  |  |  |  |  | *p^a^* |
|  | *P^c^* |  |  |  |  |  | *p^b^* |
| Heart rate, beats/min | Modified HFNC |  |  |  |  |  | *p^a^* |
|  | SOT |  |  |  |  |  | *p^a^* |
|  | *P^c^* |  |  |  |  |  | *p^b^* |
| Mean arterial pressure, mmHg | Modified HFNC |  |  |  |  |  | *p^a^* |
|  | SOT |  |  |  |  |  | *p^a^* |
|  | *P^c^* |  |  |  |  |  | *p^b^* |
| **EIT measurements** |  |  |  |  |  |  |  |
| TIV, ml | Modified HFNC |  |  |  |  |  | *p^a^* |
|  | SOT |  |  |  |  |  | *p^a^* |
|  | *P^c^* |  |  |  |  |  | *p^b^* |
| ΔEELI, ml | Modified HFNC |  |  |  |  |  | *p^a^* |
|  | SOT |  |  |  |  |  | *p^a^* |
|  | *P^c^* |  |  |  |  |  | *p^b^* |

EIT electrical impedance tomography, HFNC high-flow nasal cannula, SOT standard oxygen therapy, SpO_2_ peripheral capillary oxygen saturation, TIV tidal impedance variation, ΔEELI the changes end-expiratory lung impedance

*p^a^* for overall comparisons of differences in each group over time.

*p^b^* for overall comparisons of differences between groups over time.

*p^c^* for comparisons of differences between groups at each time point.

Table 6. Comparison of arterial blood gas parameters between the modified HFNC oxygen group and SOT groups at T0 and T4

| **Variables** | **Modified HFNC oxygen group** | **SOT group** | ***P*** |
| --- | --- | --- | --- |
| **Arterial blood gas at T0** |  |  |  |
| pH |  |  |  |
| PaO_2_, mmHg |  |  |  |
| PaCO_2_, mmHg |  |  |  |
| HCO_3_^-^, mmol/L |  |  |  |
| PaO_2_/FiO_2_, mmHg |  |  |  |
| **Arterial blood gas at T4** |  |  |  |
| pH |  |  |  |
| PaO_2_, mmHg |  |  |  |
| PaCO_2_, mmHg |  |  |  |
| HCO_3_^-^, mmol/L |  |  |  |
| PaO_2_/FiO_2_, mmHg |  |  |  |

HFNC high-flow nasal cannula, SOT standard oxygen therapy, PaO_2_ partial pressure of arterial oxygen, PaCO_2_ partial pressure of arterial carbon dioxide, HCO_3_^-^ bicarbonate, FiO_2_ fraction of inspired oxygen

Table 7. Bronchoscopic procedure and related events

| **Variables** | **All patients** | **Modified HFNC oxygen group** | **SOT group** | ***P*** |
| --- | --- | --- | --- | --- |
| **Bronchoscopic procedure** |  |  |  |  |
| Bronchoalveolar lavage, no. (%) |  |  |  |  |
| Amount of fluid instilled, ml |  |  |  |  |
| Amount of fluid recovered, ml |  |  |  |  |
| Bronchial brushing, no. (%) |  |  |  |  |
| Endobronchial biopsy, no. (%) |  |  |  |  |
| Transbronchial lung biopsy, no. (%) |  |  |  |  |
| **Events during bronchoscopy, no. (%)** |  |  |  |  |
| Agitation |  |  |  |  |
| Bronchospasm |  |  |  |  |
| Arrhythmias or tachycardia ≥ 150 beats/min |  |  |  |  |
| Hypertension (systolic BP > 180mmHg) |  |  |  |  |
| Epistaxis |  |  |  |  |
| Mucosal bleeding |  |  |  |  |
| **Events within 24h after bronchoscopy, no. (%)** |  |  |  |  |
| Transient fever |  |  |  |  |
| Pneumothorax |  |  |  |  |
| Hemorrhage |  |  |  |  |

HFNC high-flow nasal cannula, SOT standard oxygen therapy

Table 8. Clinical characteristics of patients intubated within 24 hours after bronchoscopy

| **Patients** | **Age, years** | **APACHE II score** | **Diagnosis** | **Respiratory support pre-FB** | **PaO_2_/FiO_2_ at randomization, mm Hg** | **Duration of FB, min** | **ΔEELI at T2, ml** | **Time to intubation post-FB, h** | **28-day survival status** |
| --- | --- | --- | --- | --- | --- | --- | --- | --- | --- |
| **Modified HFNC oxygen group** |  |  |  |  |  |  |  |  |  |
|  |  |  |  |  |  |  |  |  |  |
|  |  |  |  |  |  |  |  |  |  |
|  |  |  |  |  |  |  |  |  |  |
| **SOT group** |  |  |  |  |  |  |  |  |  |
|  |  |  |  |  |  |  |  |  |  |
|  |  |  |  |  |  |  |  |  |  |
|  |  |  |  |  |  |  |  |  |  |

APACHE II Acute Physiology and Chronic Health Evaluation II, PaO_2_ partial pressure of arterial oxygen, FiO_2_ fraction of inspired oxygen, ΔEELI the changes end-expiratory lung impedance, HFNC high-flow nasal cannula, SOT standard oxygen therapy

# 11. References

1. Kvale PA. Prevention and management of hypoxemia during fiberoptic bronchoscopy. Chest. 2002; 121(4): 1021-1022.
2. Du Rand IA, Blaikley J, Booton R, et al. British Thoracic Society guideline for diagnostic flexible bronchoscopy in adults: accredited by NICE. Thorax. 2013; 68 Suppl 1 :i1-i44.
3. Cracco C, Fartoukh M, Prodanovic H, et al. Safety of performing fiberoptic bronchoscopy in critically ill hypoxemic patients with acute respiratory failure. Intensive Care Med. 2013; 39(1): 45-52.
4. Agresti A, Caffo B. Simple and effective confidence intervals for proportions and differences of proportions result from adding two successes and two failures. The American Statistician. 2000, 54(4): 280-288.
5. Miettinen O, Nurminen M. Comparative analysis of two rates. Statistics in medicine, 1985, 4(2): 213-226.
6. Dong G, Li D, Ballerstedt S, Vandemeulebroecke M. A generalized analytic solution to the win ratio to analyze a composite endpoint considering the clinical importance order among components. Pharm Stat. 2016; 15(5): 430-437.
